# Methodology for a freshly engineered or cryo-preserved 3D tuberculoma bioplatform for studying tuberculosis biology and high-content screening of therapeutics

**DOI:** 10.3389/fimmu.2026.1695558

**Published:** 2026-03-20

**Authors:** Suraj B. Sable, Allison Kline, Wen Li, James E. Posey

**Affiliations:** Division of Tuberculosis Elimination, National Center for HIV, Viral Hepatitis, STD, and TB Prevention, Centers for Disease Control and Prevention, Atlanta, GA, United States

**Keywords:** 3D cell culture, granuloma model, high-throughput screening, host-directed therapy, *M. marinum*, physiological microsystems, shelf-stable, tuberculosis

## Abstract

Tuberculomas are the conglomeration of tuberculous granulomas into structurally organized three-dimensional (3D) masses that result from *Mycobacterium tuberculosis* infection and represent one of the more severe morphological forms of tuberculosis (TB). Several *in vitro* models that mimic human TB granulomas have been reported to decipher complex host-pathogen interactions and to discover new prophylactic and therapeutic interventions. They serve as ethical bridge approaches to human studies. However, these models need improvements in generating well-organized granuloma lesions, classic tuberculoma structures, and relevant microenvironments. They are impractical for screening extensive chemical and genetic libraries owing to their low throughput, limited scalability, batch-to-batch variability, and high costs. Here, we describe a ‘mycobacteria-in-spheroid’ co-culture workflow in a standard 96-well plate format that generates a robust 3D cell culture model. This model reproduces key attributes and microenvironments in human tuberculomas and can be scaled up as a high-throughput screening (HTS)-compatible bioplatform. The tuberculoma-like structures generated encompass organized, florid granulomatous foci and exhibit solid, necrotic, and cavitary morphologies. This model can be developed using freshly isolated human primary cells or a monocytic cell line with virulent mycobacteria. The platform combines the entire workflow, from generation to imaging of tuberculoma-like structures, *in situ*. It permits the serial quantitation of drug efficacy and monitoring of lesion resolution over several days to weeks following a single treatment. Additionally, we outline a methodology for adopting this workflow for cryo-preservation, enhancing its potential for commercial application. The ease of generation, pliability, cryo-shelf stability, and reproducibility of the bioplatform make it ideal for HTS applications and for implementation in discovery programs for TB and other granulomatous diseases.

## 1 Introduction

Tuberculosis (TB), caused by *Mycobacterium tuberculosis* (*Mtb*), is the leading cause of death worldwide from a single infectious agent, with over a million deaths annually (1). A key histopathological feature of TB is the formation of dynamic, spatially organized, multicellular clusters called granulomas. These macrophage-rich structures serve as sites for *Mtb* growth and dissemination, while providing environments in which infected macrophages interact with other recruited cells to “wall off” and fight the offending pathogen (2–5). One of the more severe clinical manifestations of TB is the formation of tuberculomas, which conglomerate tuberculous granulomas into well-circumscribed masses, most often in the lungs and brain, resembling cancer tumors in these organs (6–10). Tuberculomas, characterized by a severe morphological form, are present in about 5–10% of patients with pulmonary TB (11, 12). In pulmonary TB patients, granulomas are highly polymorphic and exhibit a spectrum of structures, including solid, hypoxic, necrotic, and cavitary transformations, with complex morphologies observed in lung tissue sections (13–15). Although spheroid granuloma nodules with diameters of 2–5 mm are common in the lungs, tuberculoma structures can vary from a few millimeters to over 10 cm in size (11, 14). The cavitary transformation in tuberculomas increases the risk of person-to-person transmission. Additionally, it is associated with poor treatment outcomes, relapse, and the increased likelihood of developing drug resistance in pulmonary TB (16).

Improved and shorter treatment regimens for both drug-resistant and drug-susceptible TB are urgently required to eliminate TB. Such regimens may arise from a better understanding of how new therapeutics perform across different granuloma forms and microenvironments. An increasing number of preclinical and clinical studies highlight the importance of designing novel pathogen-targeted and host-directed therapies (HDTs) that readily penetrate and act within the granuloma environment (17–21). Therapeutics that modulate host–*Mtb* interactions in granulomas can be identified using animal models and *in vitro* cell culture systems. Several two-dimensional (2D) and three-dimensional (3D) *in vitro* cell culture models that mimic nascent TB granulomas have been described in recent years using *Mtb* infection of primary human cells (22–29). Although helpful in screening a limited number of compounds and probing *Mtb*–host interactions (30–32), these models suffer from low throughput, limited tractability, and restricted scalability (33, 34). They form microgranulomas, or small granulomatous aggregates of macrophages, but lack organized lesions and relevant tuberculoma size, structures, and forms.

Consequently, physiological gradients of nutrients, oxygen, and pH, and pertinent microenvironments, present in heterogeneous TB lesions are either absent in these models or not comprehensively reproduced. Critical features such as hypoxia and *Mtb* dormancy, observed in some of these 3D models with miniature granulomas, primarily arise from the encapsulation of macrophages within microspheres or their embedding in the extracellular matrix (ECM) rather than from the granuloma structures themselves (35–37). The lack of a continuous influx of immune cells in these models makes it challenging to maintain dynamic structures and extend experiments for prolonged periods. Using animal models for such screening is costly and time-consuming, and it limits the number of compounds that can be screened in a high-containment facility. A high-throughput screening (HTS)-compatible, widely applicable bioplatform that reproduces crucial features and microenvironments in TB lesions, enabling serial multiparametric readouts of host and pathogen physiology and spatiotemporal dynamics, is highly desirable.

**Figure 1:**
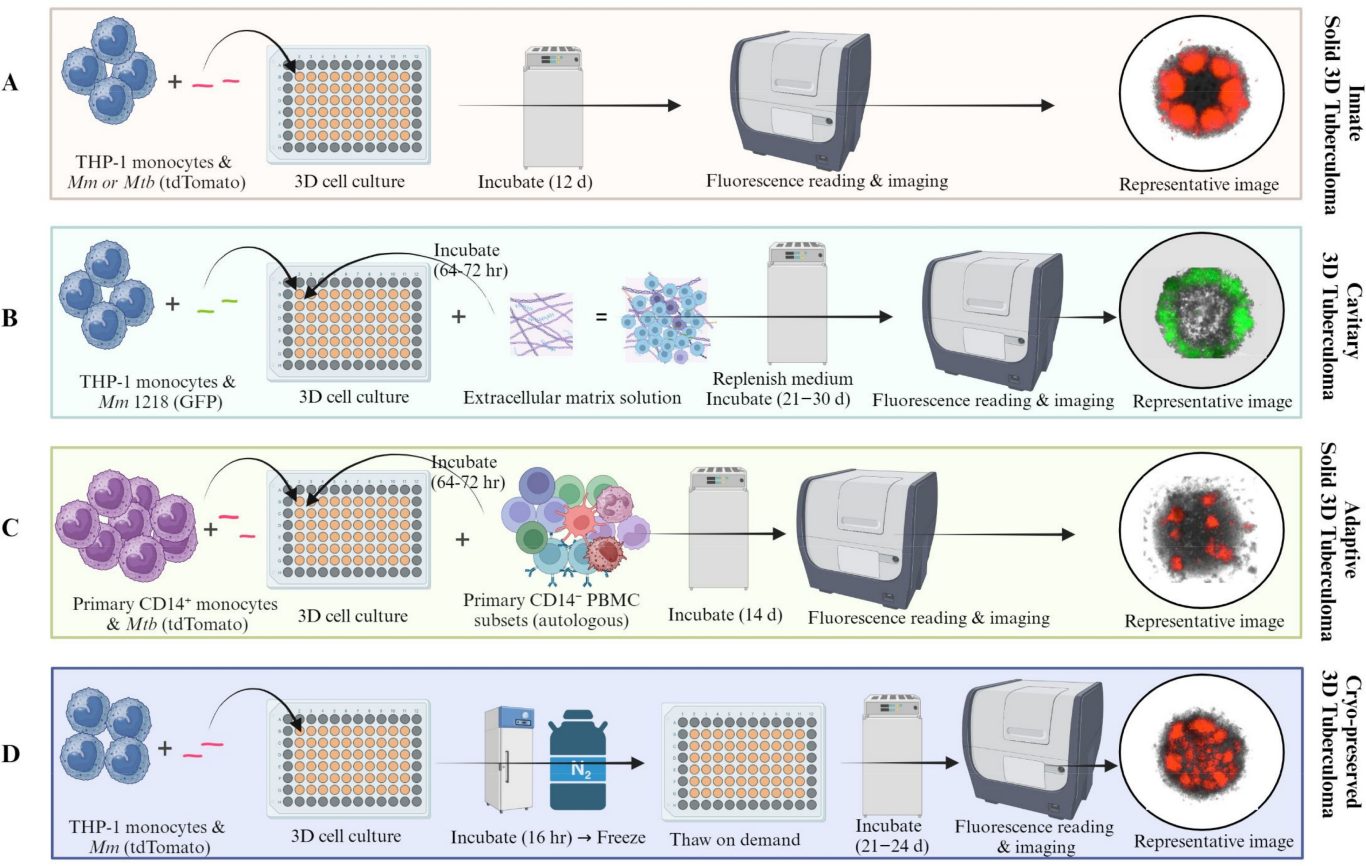
Graphical representation of the ‘mycobacteria-in-spheroid’ co-culture workflows involving human monocytes and fluorescent pathogenic mycobacteria to create a 3D tuberculoma bioplatform. The bio-protocol offers 3D human *in vitro* granuloma technology with broad applications in tuberculosis research. The 3D cell culture workflows generate **(A)** Innate solid 3D tuberculoma-like structures or **(B)** Cavitary 3D tuberculoma-like structures using immortalized human monocytes, and **(C)** Adaptive solid 3D tuberculoma-like structures using human innate and adaptive cell subsets purified from the peripheral blood mononuclear cells (PBMCs), and **(D)** Cryopreserved 3D tuberculoma-like structures using immortalized monocytes in a 96-well 3D cell culture microplate. The bioplatform does not require complex materials or specialized equipment for development and can be coupled to an automated multimode plate reader, a live-cell imager, or a fluorometer. The protocol typically spans 2–3 weeks but can be prolonged for several weeks to investigate host-pathogen dynamics or treatment response over an extended period. A versatile, tractable, and high-throughput screening-compatible platform can be used in a BSL-2 or BSL-3 laboratory to screen therapeutic libraries and be preserved for future use. A graphical overview is created in BioRender.com.

**Figure 2:**
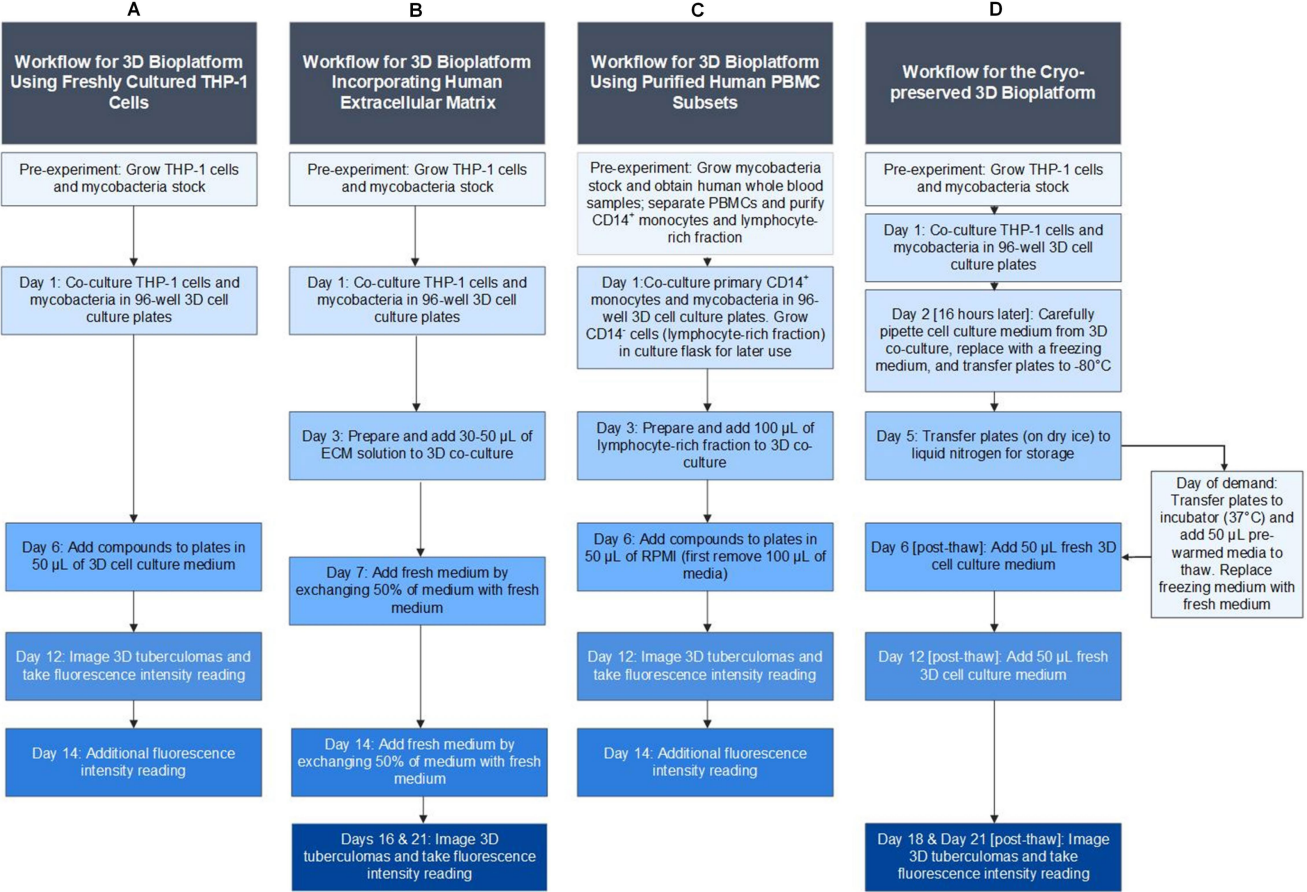
Schematic of the ‘mycobacteria-in-spheroid’ co-culture workflow types in a 96-well 3D cell culture microplate to generate a 3D human tuberculoma bioplatform. **(A)** A workflow using freshly cultured immortalized human THP-1 monocytes and pathogenic mycobacteria to generate innate, solid 3D tuberculoma-like structures. **(B)** A modified workflow of THP-1 monocytes and pathogenic mycobacteria incorporating human extracellular matrix to generate 3D tuberculoma-like structures with cavitary features. **(C)** A workflow of purified primary human blood CD14^+^ monocytes and pathogenic mycobacteria that subsequently includes lymphocyte-rich autologous PBMC subsets to generate human donor-specific nascent 3D *in-vitro* tuberculomas with innate and adaptive immune cells. **(D)** A workflow for cryo-stable 3D tuberculoma bioplatform using human THP-1 monocytes and pathogenic mycobacteria that can be frozen for future use and revived on demand to generate solid 3D tuberculoma-like structures. 3D, three-dimensional; THP-1 (Tohoku Hospital Pediatrics-1), a monocytic cell line isolated from the peripheral blood of a human leukemia child patient; ECM, extracellular matrix; and PBMC, peripheral blood mononuclear cells.

The protocol described here provides simple workflows for developing an HTS-compatible bioplatform using human monocytes and virulent *Mycobacterium* strains expressing fluorescent proteins, such as bright-red fluorescent protein (tdTomato), for fluorescence intensity and image-based assessment of drug efficacy in 3D cell-culture microplates (**Figure 1**). The ‘mycobacteria-in-spheroid’ co-cultures generated in microwells consistently form 3D tuberculoma-like structures. Since macrophages are cardinal to the core-scaffold formation that shapes host immune responses and therapeutic access in tuberculous granulomas (38), we employed human peripheral blood mononuclear cells (PBMCs) or THP-1 monocytes as macrophage sources. Methodologies for three different versions of the bioplatform, using freshly cultured THP-1 monocytes or purified primary CD14^+^ monocytes from PBMCs, are described (**Figures 2A–C**). To our surprise, human immortalized THP-1 monocytes, which possess self-renewal and recruitment capabilities, could develop structurally organized granulomatous lesions (foci) in the absence of other myeloid, lymphoid, and non-hematopoietic cells in 3D co-culture.

Furthermore, this 3D spheroid co-culture developed a range of morphological forms that imitate solid, necrotic, and cavitary tuberculomas, not previously described in existing 3D *in vitro* granuloma models. Other advantages of this platform include ease of development, increased throughput, scalability, pliability, and real-time monitoring of bacterial burden and granulomatous lesions (foci) *in situ*. In addition, we successfully used risk group 2 pathogen *M. marinum* (*Mm*) as an alternative to *Mtb* to develop this bioplatform in a BSL-2 laboratory, yielding a bioplatform comparable to that created using *Mtb*. *Mm* can cause granulomatous skin infections in humans, known as ‘swimming pool or fish tank granulomas’, through contaminated water. In zebrafish, it can develop a human TB-like disease characterized by macrophage-epithelization and tuberculous granulomas (39, 40). The *Mm*–zebrafish model has emerged as a useful *in vivo* platform for studying tuberculous granuloma formation and host-pathogen interactions (38, 41). The workflow with *Mm* can significantly reduce the staff hours required for extensive chemical or genetic screening in the BSL-3 laboratory, while also reducing safety concerns and costs.

Since the successful preservation, maintenance, and long-term storage of the screening bioplatform are highly desirable, we adapted the workflow to develop a cryo-stable version for potential commercialization that can be frozen for future use and revived on demand (**Figure 2D**). The bioplatform and techniques described have the potential to reduce the need for animal testing in high-containment facilities by improving screening capacity and efficiency, thereby saving time and resources. Although the system was developed for *Mtb*, we have provided a foundation for others to modify it for other mycobacterial and granulomatous diseases, including TB-associated co-infections and co-morbidities, such as *Mtb* infection with HIV, influenza, or SARS-CoV-2, and type II diabetes.

## 2 Materials and equipment

### 2.1 Biological materials

1. Human THP-1 cells (American Type Culture Collection (ATCC), catalog number: TIB-202)
2. Human PBMCs from whole blood of healthy, tuberculin skin test-negative donors collected in BD Vacutainer^®^ Cell Preparation Tubes (CPT™) (catalog number: 362761)
3. Fetal bovine serum (FBS), endotoxin-level tested and heat-inactivated (Atlas Biologicals, catalog number: F-0500-A). Store at -10 to -30 °C
4. *Mtb* strain H37Rv, Beijing F2, CDC1551, or Erdman expressing deep-red fluorescent protein tdTomato, *Mm* strain M expressing tdTomato, and *Mm* strain 1218 expressing green fluorescent protein (GFP)

Critical: The co-culture requires pathogenic mycobacteria (risk group 2 or 3). The attenuated *M. bovis* BCG strains lack a genomic virulence locus RD1 and are unsuitable.

### 2.2 Reagents

1. Trypan blue stain (*e.g.*, Invitrogen™, catalog number: T10282). Store at room temperature (RT)
2. Apoptosis/Necrosis kit (Abcam, catalog number: ab176750) (optional). Store at -20 °C
3. Invitrogen ImageiT Red Hypoxia Reagent (Thermo Fisher Scientific, catalog number: H10498) (optional). Store at ≤-20 °C. Stable for at least 6 months after receipt
4. Acidosis/pHrodo Intracellular pH indicator from Molecular Probes (Thermo Fisher Scientific, catalog number: P35373) (optional). Desiccate and protect from light. Store at 2–8 °C
5. Triton X-100 (*e.g.*, Sigma-Aldrich, catalog number: X-100). Store at RT
  Caution: This chemical is harmful if swallowed and causes skin irritation and severe eye damage.
6. Magnetic-assisted cell sorting (MACS) kit LS columns (Miltenyi Biotec, catalog number: 130-042-401). Store at RT
7. *MACS*^®^ CD14 MicroBeads, human CD14^+^ monocyte separation reagent (Miltenyi Biotec, catalog number: 130-050-201). Store at 2–8 °C
8. Dimethyl sulfoxide (DMSO), cell-culture grade, and endotoxin level tested (Sigma-Aldrich, catalog numbers: D2650-5×10ML and D5879). Store at RT
9. Polyvinylpyrrolidone (PVP) (MP Biomedicals, catalog number: 102786) (optional). Store at 2–8 °C

### 2.3 Media and solutions

1. RPMI 1640 medium with L-glutamine and with or without phenol red (Gibco™, catalog numbers: 11875–093 and 11835-030). Protect from light. Shelf life is 12 months from the date of manufacture. Store at 2–8 °C
2. Penicillin-Streptomycin solution, 10,000 units/ml Penicillin and 10,000 µg/ml Streptomycin antibiotics (Gibco™, catalog number: 15140-122). Shelf life is 12 months from the date of manufacture. Store at -5 °C to -20 °C
3. Sodium pyruvate solution, 100 mM (Gibco™, catalog number: 11360-070). Protect from light. Shelf life is 12 months from the date of manufacture. Store at 2–8 °C
4. HEPES buffer, 1M (Gibco™, catalog number: 15630-080). Shelf life is 24 months from the date of manufacture. Store at 2–8 °C
5. Mycobacterial liquid growth medium, Middlebrook 7H9 broth with supplements (e.g., prepared in-house, see Recipes. Middlebrook 7H9 broth dehydrated base, BD™, catalog number: 271310; Tween 80, Fisher, catalog number: BP338-500; Middlebrook albumin, dextrose, catalase (ADC) enrichment, BD, catalog number: 212352; and glycerol, Sigma-Aldrich, catalog number: G7893). Store at 2–8 °C
6. VitroCol Type 1 human collagen solution (Advanced BioMatrix, catalog number: 5007-20ml). Shelf life is 6 months from the date of receipt. Store at 2–10 °C
7. Fibronectin from human plasma, 0.1% solution (Sigma-Aldrich, catalog number: F0895). Store at 2–8 °C
8. Sodium hydroxide solution (NaOH) 0.1 M for cell culture (Advanced Biomatrix, catalog number: 5078). Store at RT
  Caution: This chemical causes skin burns and eye damage and is corrosive to metals.
9. Dulbecco’s Phosphate Buffered Saline (PBS), Calcium and Magnesium free, sterile, pH 7.4 (Gibco™, catalog number: 14190-136). Shelf life is 36 months from the date of manufacture. Store at RT
10. Cell culture-grade water, sterile (Corning™, catalog number: 25-055-CM). Store at RT
11. Phosphate buffer saline (PBS) 10× without Calcium and Magnesium (Advanced BioMatrix, catalog number: 5076-B). Shelf life is at least 6 months from the date of receipt. Store at RT
12. Red blood cell (RBC) lysis buffer, 1× eBioscience™ (Invitrogen, catalog number: 00-4333-57). Use within 6 months once opened. Store at 2–8 °C
13. Ethylenediaminetetraacetic acid (EDTA) solution (0.5M), sterile (Amresco, catalog number: E-177-500ml). Store at RT
  Caution: This chemical can cause irritation to the eyes, skin, and mucous membranes.
14. Leibovitz’s L-15 medium (Gibco™, catalog number: 11415-064) (optional). Protect from light. Shelf life is 36 months from the date of manufacture. Store at 2–8 °C
15. Cell growth medium (complete RPMI-1640 medium with antibiotics) (see Recipes)
16. 3D cell culture medium (complete RPMI-1640 medium without antibiotics) (see Recipes)
17. Middlebrook 7H9 broth (see Recipes)
18. Extracellular matrix (ECM) solution (see Recipes)
19. PBMC wash buffer (see Recipes)
20. MACS buffer (see Recipes)
21. 3D cell culture freezing mediums (see Recipes)

### 2.4 Recipes

Note: The final concentration and amount of each reagent or solution used in the recipes described below are presented in the **Supplementary Table 1–9**.

#### 2.4.1 Middlebrook 7H9 broth

To prepare 1 liter of Middlebrook 7H9 broth, dissolve 4.7 gm of 7H9 broth in 896 ml of distilled water. Add 0.05% (vol/vol) Tween-80 and 0.4% glycerol, mix well, adjust the pH to 6.8–7.0, and filter-sterilize using a 0.2 µm filter assembly. Add 10% ADC aseptically. Store at 2–8°C until use. Determine the sterility of the 7H9 broth by incubating a small volume at 37 °C for 48 hr and then at room temperature (15–25°C) for an additional 5 days before use. Stable for at least one month.

#### 2.4.2 Cell growth medium

To prepare a complete cell growth medium, supplement RPMI 1640 containing L-glutamine (2 mM) with 9.63% (vol/vol) heat-inactivated FBS, 0.87% (vol/vol) sodium pyruvate solution, 0.87% (vol/vol) HEPES buffer, and 1% (vol/vol) Penicillin-Streptomycin solution and sterile filter using a 0.2 µm Nalgene filter assembly. Store at 2–8°C until use. It is stable for at least one month.

#### 2.4.3 3D cell culture medium

To prepare a 3D cell culture medium, supplement RPMI 1640 containing L-glutamine (2 mM) with 9.73% (vol/vol) heat-inactivated FBS, 0.88% (vol/vol) sodium pyruvate solution, 0.88% (vol/vol) HEPES buffer, and filter using a 0.2 µm Nalgene filter assembly. Store at 2–8°C until use. It is stable for at least one month.

#### 2.4.4 Collagen solution and extracellular matrix (ECM) solution mixture

To prepare a collagen solution and an ECM solution mixture, slowly add 1-part chilled 10× PBS to 8 parts chilled VitroCol Type 1 human collagen stock solution (3 mg/ml), gently swirling between additions. Adjust the mixture’s pH to 7.2–7.6 with sterile 0.1M NaOH. Mix gently by pipetting up and down. Monitor the pH adjustment carefully using pH paper or a pH meter. Adjust the final volume to 10 parts with sterile cell culture-grade water. Maintain the mixture’s temperature at 2–10°C to prevent gelation. Use the solution within 16 hr. Add 40 µl of human fibronectin (0.1%) solution to the 10 ml collagen mixture before use. Mix gently by pipetting up and down.

#### 2.4.5 PBMC wash buffer

To prepare the PBMC wash buffer for washing PBMCs after RBC lysis, supplement PBS without calcium and magnesium, pH 7.2–7.4, with 10% (vol/vol) heat-inactivated FBS and 2 mM EDTA. Filter the buffer using a 0.2 µm Nalgene filter assembly. Prepare the buffer on the day of use and keep it cold (2–8°C).

#### 2.4.6 MACS magnetic labeling and column elution buffer

To prepare the MACS buffer, supplement PBS without calcium and magnesium, pH 7.2–7.4, with 0.5% (vol/vol) heat-inactivated FBS and 2 mM EDTA. Prepare the buffer on the day of use and keep it cold (2–8°C).

#### 2.4.7 3D cell culture freezing media

in section a, b and c, remove the line breaks between paragraphs and indent the first line as normal.

To prepare the freezing medium, supplement RPMI 1640-based complete 3D cell culture medium (Recipe 3) with 5% (vol/vol) DMSO. Prepare before use and filter sterilize using a 0.2 µm Nalgene filter assembly.

b. FBS with DMSO (optional)

To prepare the freezing medium, supplement a heat-inactivated FBS with 5% (vol/vol) DMSO. Prepare before use and filter-sterilize using a 0.2 µm filter.

c. L15 medium with cryoprotectants (optional)

This freezing medium contains equal volumes of serum freezing medium “A” (2×) and DMSO freezing medium “D” (2×). First, add 100 µl of freezing medium “A” to the microwells containing the 3D cell culture, and then add 100 µl of freezing medium “D” before transferring the microplates to a -80°C freezer.

1. Serum freezing medium “A” (2×)

To prepare PVP-10× stock, add 10% PVP (wt/vol) to 1× HEPES-buffered saline. Stock 10× HEPES-buffered saline contains 70.7 gm of sodium chloride, 17.0 gm of glucose (dextrose), 2.0 gm of potassium chloride, 19.4 gm of sodium phosphate monobasic 2H_2_O, 47.6 gm of HEPES buffer, and 0.01 gm of phenol red in up to 1000 ml of cell-culture-grade water and 1× HEPES-buffered saline is prepared by adding 900 ml of cell culture grade water in 100 ml of 10× HEPES-buffered saline. To prepare 1000 ml of serum freezing medium “A” (2×), supplement 484 ml of L15 medium with 16 ml of 1M HEPES buffer, 300 ml of heat-inactivated FBS, and 200 ml of PVP-10× stock and filter-sterilize using a 0.2 µm filter.

2. DMSO freezing medium “D” (2×)

To prepare 1000 ml of DMSO freezing medium “D” (2×), add 16.02 ml of 1M HEPES buffer and 150.63 ml of DMSO to 833.3 ml of L15 medium, and then filter-sterilize using a 0.2 µm filter. The serum freezing medium “A” and DMSO freezing medium “D”, stored in glass bottles at -20°C, are stable for at least one month.

### 2.5 Laboratory supplies

1. Nalgene^®^ Rapid-Flow™ single-use vacuum filter units, sterile (500 ml, with 0.2 µm membrane) (*e.g*., Thermo Fisher Scientific, catalog number:5660020)
2. Reagent reservoirs, sterile, disposable (*e.g.*, Aquafill, catalog number: S-5501080)
3. Micropipette tips with filters (various volumes), sterile, disposable (*e.g.*, Rainin™, catalog numbers: 30389257, 30389272, 30389276, and 30389274)
4. Serological pipettes (various volumes) (*e.g.*, Pyrex, catalog numbers: 7077-5N and 7077-10N)
5. Cell culture flasks, 75 cm^2^, 150 cm^2^ and 175 cm^2^, sterile (Corning™, catalog numbers: 430720U, 431465, and 431080)
6. Falcon™ conical centrifuge tubes, 15 and 50 ml, sterile (Corning™, Falcon^®^, catalog numbers: 352097 and 352098)
7. Microcentrifuge tubes (various volumes, sterile) (*e.g.*, GreenTree Scientific, catalog number: T5040G and LabSource, catalog number T56-950)
8. Tube holder or rack (*e.g.*, Fisher Scientific, catalog numbers: 21-200-285, 03-448-17, 21-402-18)
9. Syringe fitted with a needle (25 or 27G), sterile (*e.g.*, Becton Dickinson (BD), catalog number: 309626)
10. 3D cell culture plates (Corning^®^ Spheroid Microplates, catalog number: 4515)
  Note: We tested microplates from several different vendors.
  Critical: Corning^®^ Ultra-Low Attachment (ULA) Spheroid Microplates were optimal for generating 3D tuberculoma-like structures described here. This 96-well microplate has an opaque black body that shields each optically clear microwell from well-to-well crosstalk. The round-bottom microwells have a hydrophilic, biologically inert surface. They are coated with a covalently bonded, non-ionic, neutrally charged hydrogel that minimizes activation and cell adhesion to the microwell surface, enabling uniform and reproducible 3D tuberculoma-like structure formation. Another helpful alternative is S-BIO PrimeSurface^®^ 3D Culture Spheroid Plates (S-BIO, catalog number: MS-9096UZ)
11. Countess™ Cell Counting Chamber Slides (Invitrogen™, catalog number: C-10283) or Neubauer cell counting chamber (*e.g.*, Millipore Sigma, Bright-Line hemocytometer, catalog number: Z359629)
12. Mycobacterial culture bottle (e.g., 490 cm^2^ sterile roller bottle, Corning^®^, catalog number: 430195)
13. Mycobacterial culture media plates (*e.g.*, Middlebrook 7H10 agar plates without antibiotics and supplemented with oleic acid, albumin, dextrose, and catalase (OADC) enrichment 10% (vol/vol) and glycerol 0.5% (vol/vol) prepared in-house. Middlebrook 7H10 Agar, BD Difco™, catalog number: 262710; Middlebrook OADC, BD, catalog number 212351; and Glycerol, Sigma-Aldrich, catalog number: G7893)
14. Bacterial cell spreaders (*e.g.*, Fisher Scientific, catalog number: 14-665-230)
15. Cell scraper (e.g., Costar™, catalog number: 3010)
16. Petri dish (*e.g.*, Falcon™, catalog: 351029)
17. Assay block 96 well, 2 ml capacity, sterile (Corning™, catalog: 3960)

### 2.6 Equipment

1. Class II Type A2 biological safety cabinet (BSC) (*e.g*., Nuaire, Model: NU-543-600)
2. Micropipettes (various volumes) (*e.g.*, Rainin™, catalog numbers: Pipet-Lite LTS Pipettes L-20XLS, L-200XLS, L-1000XLS, L-5000XLS, and L8-200XLS)
3. Novaspec II spectrophotometer (Amersham Pharmacia Biotech) or equivalent
4. Aerosolve^®^ canisters or equivalent
5. Cell culture incubator, set at 37°C with 5% CO_2_ and >95% humidity (Forma Scientific, Model: 3110)
6. Microcentrifuge for microtubes (Eppendorf, Model: 5430 R)
7. Centrifuge for 15- or 30-ml conical tubes (Eppendorf, Model: 5810 R)
8. Cell counting equipment (Thermo Fisher Scientific, Model: AMQAX1600 Countess II or AMQAX2000 Countess III)
9. pH meter or pH paper (*e.g.*, Fisher brand, catalog number: 13-640-510)
10. Cytation 5 microplate fluorescence reader and cell imager or equivalent with optional CO_2_ source (i.e., CO_2_ controller) (Agilent/BioTek, Model: CYT5MPV with GEN5PRIME and GEN5SPOT)
  Note: A fluorescence plate reader and cell imager other than Cytation 5 can be used.
11. MidiMACS™ Separator (Miltenyi Biotec, catalog number: 130-042-302) and MACS Multi-Stand (Miltenyi Biotec, catalog number: 130-042-303)
12. Water bath (Precision 180 series)
13. Refrigerator, set at 2 to 8°C
14. Freezer, set at -80°C (range -75 to -85°C)
15. Liquid nitrogen storage for cell culture (temperature range -165 to -196°C)
16. Autoclave

### 2.7 Software

1. Gen5 V3.08 with Spot Counting add-on module (Agilent/BioTek, https://www.biotek.com)
  Note: Gen5 Image+ software controls the operation of the Cytation 5 for photomultiplier tube (PMT)-based microplate reading and automated digital microscopy.
2. GraphPad Prism V9.3 (GraphPad Prism by Dotmatics, https://www.graphpad.com)

## 3 Methods

### 3.1 ‘Mycobacteria-in-spheroid’ co-cultures and 3D tuberculoma bioplatform

The workflows for generating ‘mycobacteria-in-spheroid’ co-cultures and a freshly engineered or cryopreserved 3D tuberculoma bioplatform are described in detail below, including step-by-step procedures, their application for therapeutic screens, and the corresponding readouts. Essential information regarding mycobacterial cultures and frozen stocks, human primary and immortalized cell cultures, 3D ‘mycobacteria-in-spheroid’ co-cultures, tuberculoma bioplatform, antibiotic or chemical compound treatment, and characterization of microenvironments in the 3D cell cultures using fluorescent probes are described in the workflows. These methods are alternatively summarized in the **Supplementary Material** and presented as the **Supplementary Methods**.

### 3.2 Statistical analysis

The Mann-Whitney test was used to assess differences between two groups, while differences among three or more groups were assessed using the one-way ANOVA and the nonparametric Kruskal–Wallis test with Dunn’s *post-hoc* test (GraphPad Prism V9.3). A p< 0.05 was considered statistically significant, and symbols *, **, ***, and **** in the figures indicate p< 0.05,< 0.01,< 0.001, and< 0.0001 respectively. The quality of the high-content drug screening assay in the 3D tuberculoma bioplatform using a co-culture of THP-1 monocytes and *Mm* was determined using the Z’ statistic. The Z’-factor describes how well-separated the positive and negative controls are in the HTS assay without the intervention of test compounds. The Z’-factor was calculated by performing the assay across a batch of 8–10 different 3D cell culture microplates as follows:


$$Z^{\prime }=1-\;\frac{\left(3\sigma_{c-}+3\sigma_{c+}\right)\;}{\left|{µ}_{c-}-{µ}_{c+}\right|}$$

where $\sigma_{c-}$ is the standard deviation of the untreated or DMSO control wells, $\sigma_{c+}$ is the standard deviation of the rifampicin or nitazoxanide control wells, ${µ}_{c-}$ is the mean of the untreated or DMSO control wells, and ${µ}_{c+}$ is the mean of the rifampicin or nitazoxanide control wells. A Z’ value between 0.5 and 1 is considered excellent, a value between 0 and 0.5 is acceptable, and a value less than 0 indicates that the assay is unlikely to be suitable for HTS applications.

### 3.3 Step-by-step procedures

#### 3.3.1 Workflow A for 3D tuberculoma bioplatform using freshly cultured THP-1 monocytes

Note: This optimized workflow outlines the steps for developing a tuberculoma bioplatform in a 96-well 3D cell culture microplate using a simple co-culture of THP-1 monocytic cells and fluorescent pathogenic mycobacteria for high-content screening (HCS) of potential therapeutics. The typical workflow for screening test compounds, small molecules, or biologics spans 14 days. Depending on the experiment’s goal, the workflow can be extended beyond two weeks if the microwells are replenished with fresh culture medium and THP-1 cells. Using the *Mm* 1218 strain and carefully exchanging 50% of the medium in microwells with fresh medium once every seven days, we have successfully cultured the ‘mycobacteria-in-spheroid’ model for up to 50 days.

Caution: All work involving the handling of virulent *Mtb* strains (risk group 3 organisms) should be performed in a BSL-3 laboratory under a Class II BSC, wearing appropriate personal protective equipment (PPE). If the organism of interest is *Mm* (risk group 2), the experiment is conducted in a BSL-2 laboratory under a Class II BSC, wearing appropriate PPE.

##### **Pre-experiment.** Grow bacterial stocks and THP-1 cells

1. Prepare mycobacterial frozen stocks

Note: We tested *Mtb* and *Mm* strains that expressed red fluorescent protein tdTomato for this workflow. In some experiments, *Mm* strain 1218 GFP was used. The strains used in the study are described in the **Supplementary Methods**.

a. Inoculate 5 ml of 7H9 broth with 100–150 µl of strain (from a strain collection stored at −80 °C) in a 25×150 mm glass culture tube (30 ml-capacity) and incubate at 30 °C (*Mm*) or 37 °C (*Mtb*) for 5–10 days.

b. Measure the inoculum’s optical density at 600 nm (OD_600_). When the OD_600_ reaches 0.6–1.0, add the 5 ml inoculum to 150 ml of 7H9 broth in a 490 cm^2^ sterile culture bottle.

c. Incubate the culture at 30 °C (*Mm*) or 37 °C (*Mtb*) for 10–12 days, with daily manual shaking, until the OD_600_ reaches 1.0–1.5, as measured by the Novaspec II spectrophotometer.

d. Pellet cultures by centrifugation at 4000× *g* for 30 min at room temperature. Remove the supernatant by decanting or pipetting and then resuspend the pellet in 10 ml of fresh 7H9 broth (Recipe 1).

e. Aliquot the culture at 1.0 ml per cryovial and store at −80 °C. Use the 7H9 broth supplemented with appropriate antibiotics to prepare stocks of recombinant fluorescent strains.

f. Thaw an aliquot seven days after freezing and sub-aliquot into the 100 µl working stocks. Determine the viable count on 7H10 agar without antibiotics by incubating at 30 °C for 12–15 days (*Mm*) or 37 °C for 28–30 days (*Mtb*).

2. Grow THP-1 cells in a cell growth medium (see Recipe 2)

Note: Follow ATCC’s handling, maintenance, and initial growth procedures.

a. Seed 175 cm^2^ (T175) tissue culture flask containing pre-warmed (37 °C) 50 ml cell growth medium containing antibiotics (Recipe 2) with 4×10^6^ live THP-1 cells from the ongoing cell culture.

b. Place the tissue culture flask in a cell culture incubator set to 37 °C with 5% CO2 and >95% humidity to continue THP-1 cell growth for seven days.

**Day 1**. Development of 3D ‘mycobacteria-in-spheroid’ co-culture

1. Preparing THP-1 cell suspension for 3D cell culture

a. Aliquot 3D cell culture *medium* (see Recipe 3) in 50 ml conical tubes.

Critical: Verify the sterility of the medium before use.

b. Prewarm 3D cell culture medium in the cell culture incubator at 37 °C for 1 hr.

c. Count THP-1 cells cultured in a growth medium containing antibiotics (Recipe 2) and assess cell viability by the trypan blue dye exclusion method.

Note: A final concentration of 1×10^6^ live cells/ml is required.

Critical: Cell viability must be ≥95% after the last wash. Use a fully dissolved trypan blue dye to avoid interference with automated cell counting caused by the dye precipitates. We used an automatic cell counter, Countess II or III, for cell counting. Cells may be pooled from two or more 150 cm^2^ cell culture flasks cultured in the same batch and subjected to cell count and viability assessment. THP-1 cells cultured in two or more flasks will be required for extensive experiments that require >4 microplates.

d. Centrifuge cells for 6 min at 200−250× *g* at room temperature (15–20 °C) in 50 ml conical tubes and discard the supernatant without disturbing the cell pellet.

e. Resuspend the cell pellet in 15 ml of prewarmed (37°C) 3D cell culture medium (Recipe 3) and gently mix using a pipette. Centrifuge the cells again for 6 min at room temperature at 200–250× *g* and discard the supernatant.

f. Repeat this washing step twice more.

g. After the third wash, resuspend the cell pellet in 15 ml of prewarmed (37 °C) 3D cell culture medium, gently mix with a pipette, and let the cell suspension rest for 30 min at room temperature.

Note: This step is incorporated to exchange and remove antibiotics that are pinocytosed by THP-1 cells during culture in flasks containing antibiotic-containing growth medium.

h. Centrifuge cells for 6 min at room temperature at 200–250× *g* and discard the supernatant. Resuspend the cell pellet in 10 ml of prewarmed (37°C) 3D cell culture medium and gently mix using a pipette.

i. Perform the final cell count and assess cell viability using the trypan blue dye exclusion method.

j. Adjust the cell concentration to 1×10^6^ live cells/ml using a 3D cell culture medium.

Note: Each microplate requires a precise 6 ml of THP-1 cell suspension. Cells are not dispensed in the microwells on the periphery of the plate to avoid an “edge effect.” To mitigate evaporation, the outer wells are filled with sterile 3D cell culture medium or cell culture-grade water.

2. Preparing *Mtb* H37Rv (tdTomato) or *Mm* M (tdTomato) suspension for 3D cell culture

a. Transport the cryovial containing the frozen stock of *Mtb* H37Rv (tdTomato) or *Mm* M (tdTomato) in an Aerosolve^®^ (transport) canister from a storage freezer to the BSC at the appropriate biosafety level. Place the cryovial in a tube holder in the BSC.

b. Transfer 900 µl of 3D cell culture medium into a microtube containing 100 µl of mycobacterial working stock and mix well.

c. Centrifuge at 5000× *g* for 30 min at 8 to 10 °C. When finished, carefully discard the supernatant using a pipette, taking care not to disturb the mycobacterial pellet.

d. Repeat this wash step using a new 1 ml aliquot of 3D cell culture medium and resuspend the pellet in 1 ml of pre-warmed (37 °C) 3D cell culture medium.

e. Pass the mycobacterial suspension through a 25–27G needle attached to a 1 ml syringe 10 to 20 times to obtain a single-bacterial-cell suspension.

Note: Perform this step immediately before infecting THP-1 cells to prevent bacterial aggregation.

CAUTION: DO NOT CAP THE NEEDLE ON THE SYRINGE. Carefully place the syringe with a needle in the sharps-disposal container.

f. Transfer the required amount of mycobacterial single-cell suspension into a sterile reservoir. Dilute mycobacterial suspension using a volume of prewarmed 3D cell culture medium needed to reach the intended multiplicity of infection (MOI) and to make a necessary volume of bacterial inoculum for THP-1 cell infection in microplates.

Note: Our experiments are routinely performed using five microplates. We typically prepare 35 ml each of THP-1 cell suspension and mycobacterial suspension (infection inoculum) in sterile reservoirs. During the transfer process into microwells, we frequently mixed the inoculum by pipetting up and down to maintain a uniform single-cell suspension and prevent mycobacteria from settling in the reservoir and clumping together. MOI was calculated as the input CFU divided by the number of monocytes added per well. We did not wash monocytes after infection and avoided using aminoglycoside antibiotics to kill extracellular mycobacteria, if any. Pinocytosed aminoglycosides can reach macrophage phagosomes and contribute to cells’ antimicrobial activity (42).

Critical: The ideal MOI varies with the *Mycobacterium* species or strain used in the 3D co-culture and is determined experimentally. Refer to the Results and Data Analysis sections for the optimal MOI of *Mm* and *Mtb* strains used in the 3D co-culture. We used the optimized low MOI of 0.008 (range, 0.006–0.012) for co-cultures using *Mm* M (tdTomato).

3. Preparing 3D microplates for 3D cell culture

a. Label Corning^®^ 3D spheroid microplates with plate number, date, experiment, and performer’s name. Mark the peripheral boundary wells. Refer to the Data Analysis section for a schematic of the plate used in the compound screening assay.

b. Add 100 µl of THP-1 cell suspension (1×10^5^ live cells) into each microwell, except those on the periphery.

Note: Use six micropipette tips fitted on a multichannel pipette. Micropipette tips are angled against the walls of the wells during cell-suspension dispensing. Therefore, avoid touching the micropipette tips to the bottom of the wells.

c. Add 100 µl of the mycobacterial suspension to each microwell, except those on the periphery.

Note: Control wells containing THP-1 cells can be kept without mycobacterial infection, depending on the experimental goal. Add 100 µl of 3D cell culture medium to these wells in place of the bacterial suspension.

d. Mix thoroughly up and down at least three times, without touching the bottom of the microwell, to obtain a homogeneous suspension of THP-1 and mycobacteria. Avoid bubble formation during mixing.

e. Fill the peripheral microwells with 250–300 µl of sterile 3D cell culture media or cell culture-grade water using a multichannel micropipette.

f. Place the microplates in a cell culture incubator set at 37 °C with 5% CO_2_ and >95% humidity to continue the co-culture experiment and generate 3D ‘mycobacteria-in-spheroid’ structures in ULA round-bottom microwells.

g. Determine the actual CFU count in the 100 µl of mycobacterial suspension by plating dilutions onto Middlebrook 7H10 agar plates. Incubate agar plates at 30 °C for *Mm* M (tdTomato) for 12–14 days and 37 °C for *Mtb* H37Rv (tdTomato) for 3–4 weeks.

**Day 6**. Addition of test compounds, small molecules, or other therapeutics

1. Preparing and adding drug dilutions for screening in the 3D bioplatform

a. Prepare a dilution of test drugs in a pre-warmed (37 °C) 3D cell culture medium.

Note: The optimal drug concentration can be experimentally determined. We routinely performed screening experiments at a standard concentration of 20 µM of drug per microwell. In addition, we usually tested six different concentrations, ranging from 20 µM to 0.625 µM, for selected drugs (using 2-fold serial dilution). We performed drug dilutions in 96-well, 2-ml capacity, sterile assay blocks. For screening extensive compound libraries, stock compound solutions (10 mM) in DMSO were obtained from the commercial vendors, aliquoted, and stored at -20 °C or the recommended temperature. To get a final concentration of 20 µM of test compound per microwell, 10 µl of stock compound (10 mM) was diluted in 990 µl of pre-warmed (37 °C) 3D cell culture medium, and 50 µl of this diluted compound solution was added to the microwells containing spheroids in 200 µl of the 3D cell culture medium.

b. Observe the granulomatous lesion formation in the 3D ‘mycobacteria-in-spheroid’ co-culture using a manual mode in Cytation 5 imager or an inverted microscope (optional).

Note: Florid granuloma lesions (foci) begin to develop between days 5 and 6 in the 3D spheroid infected with the optimized low MOI of *Mm* M and *Mtb* H37Rv. The granulomatous lesions grow over time and become structurally organized. We added test drugs on day 6.

c. Slowly add the diluted drug in a 50 µl volume to the microwells without disturbing the 3D spheroids. Keep a minimum of three technical replicates for each test drug and include appropriate positive control (a known effective antibiotic or HDT drug) and negative control (medium alone [no-drug added] and drug carrier) wells in each microplate.

Note: The final volume of the medium in the microwell after drug treatment will be about 250 µl. Negative control wells will receive 50 µl of 3D cell culture medium. Critical: Pipette tips are positioned against the walls of microwells while the drug solution is slowly dispensed to minimize disturbance of the 3D spheroids and granuloma lesions formed. Please refer to the **Data analysis** section for more information about positive and negative controls used in our drug screening assay.

**Day 12**. Fluorescence intensity reading and imaging to assess antitubercular drug efficacy

1. Reading microplates to detect fluorescence intensity as a measure of bacterial burden

a. Place the microplate with lid (without the bottom plate-stand) into the Cytation 5 multi-mode plate reader, previously set to 30 °C or 37 °C and 5% CO_2._

Note: A Cytation 5 combines automated digital widefield microscopy with conventional multi-mode microplate detection to provide phenotypic cellular information and well-based quantitative data. In Gen5 software, ensure that the appropriate plate type (e.g., Corning ULA round well bottom) is selected and the vessel’s bottom elevation is defined. The fluorescence intensity in the co-culture can be measured in the absence of CO_2_ in the Cytation 5 plate reader. We performed *in situ* fluorescence readings and imaging at 30 °C, without a CO2 source and controller, on the *Mm* co-culture. We tested auto-gain and several fixed-gain readings in the microplate with 3D spheroids and found the auto-gain reading suitable for a compound screening assay. For fluorescence intensity readings in the 3D spheroids generated, the ‘top’ reading of the microplate is more optimal than the ‘bottom’ reading. A microplate fluorimeter can serve as a cost-effective alternative to a multimode fluorescence reader. The optimal time to read fluorescence intensity or imaging after drug treatment for the workflow using *Mm* M (tdTomato) is day 12. The optimal time point for measuring fluorescence intensity or imaging after drug treatment should be experimentally determined for the mycobacterial strain.

b. Read the tdTomato fluorescence intensity using the fluor-specific fluorescence intensity reading protocol developed, validated, and stored on the computer attached to Cytation 5.

c. To read fluorescence intensity and growth of mycobacteria expressing tdTomato in the Corning 96-well 3D cell culture plate, follow the four steps below.

i. Open the Gen5 software. Next, select the ‘Experiments’ tab on the left panel in the ‘Task Manager’ screen.

ii. On the right-hand side of the screen, choose ‘Create using an existing protocol’ and select the pre-developed and saved tdTomato fluorescence intensity reading protocol. The ‘Task Manager’ screen will be closed. The parameters of the tdTomato fluorescence reading protocol are listed in **Table 1**.

**Table 1:** Fluorescence intensity reading parameters.

Fluorescence intensity reading parameters	
Plate Type	Corning 96-well round-bottom ULA 3D spheroid plate with lid
Temperature	30 °C to 37 °C
Read Method	Fluorescence intensity
Read Type	Endpoint
Optics Type	Monochromators
Fluorophore	tdTomato
Excitation	588 nm
Emission	633 nm
Optics Position	Top
Gain	Auto
Read Speed	Normal
Read Height	7.00 mm
Selected Wells	B2 to G11

iii. Click the green ‘Read New’ button on the top panel of the Gen5 software. The software will prompt you to save the experiment name and other details in a specific location (directory) on your computer.

iv. After saving the experiment information, the software proceeds to read the fluorescence intensity in the pre-selected microwells of the plate.

d. Export the fluorescence intensity data from Gen5 as an Excel file and save it on the computer for data analysis.

2. Automated plate imaging using Cytation 5

a. Place the microplate with the lid into a Cytation 5 set at 30 °C or 37 °C and 5% CO_2_.

b. Capture images using the optimized protocol and the parameters listed in **Table 2**.

**Table 2:** Automated fluorescence imaging parameters.

Automated fluorescence imaging parameters		
Objective	2.5× FL Zeiss	
Plate Type	Corning 96-well round-bottom ULA 3D spheroid plate with lid	
Temperature	30 °C to 37 °C	
Read Method	Image	
Read Type	Endpoint	
Optics Type	Sony Mono CCD Camera	
Brightfield Filter	Total spheroids	
Texas Red Filter	*Mm* M or *Mtb* strain expressing tdTomato	
Fluorescence Excitation	586 nm	
Fluorescence Emission	647 nm	
Selected Wells	B2 to G11	
Channel	Brightfield	Texas Red (586,647)
LED	5	10
Integration Time	137	1000
Gain	1.45	15.6
Montage Type	2 × 2	2 × 2

Note: The ‘mycobacteria-in-spheroid’ co-culture imaging was carried out to generate quality images of 3D tuberculoma-imitative structures and assess drug efficacy in reducing fluorescent mycobacterial growth and resolution of granuloma lesions (foci). We used a 2.5× objective and a 2×2 image montage to image the entire well. A brightfield imaging channel was used to capture total spheroid images, and a red fluorescence channel (Texas red) was used to capture mycobacteria expressing tdTomato.

c. To image a 3D cell culture microplate, follow the steps below.

i. Open the Gen5 software. Select the ‘Experiments’ option in the ‘Task Manager’ window.

ii. On the right-hand side of the screen, choose ‘Create using an existing protocol’ and select the pre-developed 3D spheroid imaging protocol. The ‘Task Manager’ screen will be closed. The complete parameters of the imaging and analysis protocol are listed in **Tables 2**, **3**, **4**, **5**, **6**.

**Table 3:** Image stitching parameters.

3D image stitching parameters	
Registration Channel	Texas Red 586, 647
Montage Size	2326 × 1718 (7.62 Mb)
Fusion Method	Linear Blend

**Table 4:** Z-stacking parameters for 3D imaging.

3D imaging Z-stacking parameters	
Focus Method	Fixed focal height (1551 µM)
Number of Slices	11 (Images taken through the structure)
Step Size	68 µm
Sample Thickness	680 µm
Images Below Focus Point	0
Stacking Method	Focus stacking
Channel	Brightfield/Texas Red
Size of Max. Filter	11 pixels
Top Slice	11
Bottom Slice	1

**Table 5:** Cellular analysis parameters (spheroid analysis).

3D spheroid cellular analysis parameters [for spheroid analysis]	
Primary mask criteria (on brightfield channel)	
Threshold	5000
Background	Light
Split Touching Objects	Checked
Fill Holes in Masks	Checked
Min. Object Size	1500 µm
Max. Object Size	4000 µm
Include Primary Edge Objects	Unchecked
Analyze Entire Image	Checked
Advanced detection options	
Rolling Ball Diameter	Auto
Image Smoothing Strength	12
Evaluate the Background On	15% of the Lowest Pixels
Primary mask criteria (on texas red channel)	
Background	Dark
Measure within a Primary mask	Checked and “Use Primary Mask”
Measure within a Secondary mask	Unchecked
Count Spots	Checked
Min size	125 µm
Max size	2500 µm
Advanced options	Determined by plate fluorescence
Calculated metrics	
Metric of Interest	Cell Count
	Object Size (Spheroid)
	Object Area (Spheroid)
	Object Sum Area (Spheroid)
	Object Spot Count (Lesions)
	Object Sum Spot Count (Lesions)
	Object Spots Area (Lesions)
	Object Spots Sum Area (Lesions)
	Lesion Area Ratio (Percent Area Affected) *

*Custom metric of interest: Lesion Area Ratio = Object Spots Sum Area/Object Area.

**Table 6:** Cellular analysis parameters (lesion analysis).

3D spheroid cellular analysis parameters [for lesion analysis]	
Primary mask criteria (on Texas red channel)	
Threshold	Auto-45
Background	Dark
Split Touching Objects	Checked
Fill Holes in Masks	Unchecked
Min. Object Size	150 µm
Max. Object Size	1500 µm
Include Primary Edge Objects	Unchecked
Analyze Entire Image	Unchecked
Advanced detection options	
Rolling Ball Diameter	1000 µm
Image Smoothing Strength	5
Evaluate the Background On	5% of the Lowest Pixels
Calculated metrics	
Metric of Interest	Cell Count (Lesions)
	Object Size (Lesions)
	Object Area (Lesions)
	Object Sum Area (Lesions)

iii. Click the ‘Read New’ button on the software’s top panel. The software will prompt you to save the experiment name and details on your computer.

iv. After saving the experiment information, the software will automatically start imaging the preselected microwells in the plate.

Note: We used montage imaging, image stitching, and Z-stacking in experiment mode in Gen5 to capture 3D tuberculoma images. To capture the entire 3D tuberculoma-like structure (1800–2300 µm in diameter) that spans outside the field of view of the objective, a montage image capture mode was used to capture four image segments (tiles) in the microwell. Individual tiles in the montage are stitched together to form a single, whole image. The ‘auto for stitching’ method was used to capture the montage. The 3D tuberculoma-like structure exists within a range of Z-planes, and multiple images (slices) must be acquired by moving the objective along the Z-axis (or focal) planes. Therefore, we used the Z-stacking imaging procedure within Gen5 by selecting “Image Z-stack.” Multiple automated image slices were captured below and above the focal plane to ensure that the 3D tuberculomas, cells, and granuloma lesions were imaged at the proper Z-height. After the Z-stacked images are captured, a projection of the Z-stack is created by performing a “Z Projection” step and utilizing the focus-stacking algorithm to produce a final composite image. For image stitching and Z-projection, Gen5 Image+ software is required. Each well takes approximately 2 min to complete the imaging process using the described parameters.

d. Use the parameters listed in **Table 3** to stitch the individual tiles into a single stitched image.

e. For Z-stacking the individual slices to generate a single stacked image, use the parameters listed in **Table 4**.

3. Cellular analysis of 3D tuberculoma images in Gen5

a. Perform the cellular analysis of projected images using the parameters listed in **Table 5**, **6**.

Note: We used cellular analysis to determine the diameter and size of 3D tuberculoma-like structures, the number of encompassing granulomatous lesions, and the area affected by bacterial growth and lesions in the tuberculoma-like structures.

**Day 14**. Additional fluorescence intensity reading of microplates with 3D culture

Note: For relatively slow-growing, virulent *Mtb* strains, such as H37Rv, Beijing F2, CDC1551, or Erdman (tdTomato), we performed additional fluorescence intensity readings of the co-cultures on day 14. Although fluorescence intensity readings on day 12 provided pertinent information about the efficacy of the test drugs, the separation of relative fluorescence units (RFU) between test (or positive control) and negative control wells was comparatively better on day 14 for the workflow using *Mtb* strains. We routinely performed drug screening experiments using five microplates, but used up to 12 microplates in some experiments. For experiments with more than five microplates, imaging can be initiated on day 12, with the remaining plates imaged on the following day.

1. Read the tdTomato fluorescence intensity using the parameters described above.

Note: The workflow described above generates a solid tuberculoma-like structure in each microwell with 3D co-culture (See Results). The resulting 3D tuberculoma-like structures are highly uniform in size distribution, bacterial growth, and the granulomatous lesions (foci) encompassed. The platform, in 96-well format, can be employed as an HCS and imaging assay platform for anti-TB drug discovery (see the Data processing and analysis section).

#### 3.3.2 Workflow B for 3D tuberculoma bioplatform incorporating human extracellular matrix

Note: This workflow details the steps required to develop a bioplatform using a co-culture of THP-1 cells and a pathogenic mycobacterial strain in the presence of physiologically relevant human ECM components. ECM contributes to the architecture of tuberculous granulomas in the human lungs, and its immunopathological destruction leads to cavity formation in TB patients (4, 16, 43). In addition, collagen-rich ECM regulates macrophage survival in 3D *in vitro* granuloma models (26). Still, the human 3D *in vitro* granuloma model with cavitary transformations has yet to be described. Advances in 3D *in vitro* granuloma models that develop cavitary features will enable investigations into the drivers of cavitation and pharmacological interventions to identify HDT drugs that can prevent or treat *Mtb*-induced tissue destruction and immunopathology. The 3D tuberculoma model described here develops cavity-like features in spheroid structures. The pre-experiment steps for this workflow include growing *Mm* 1218 GFP stocks and THP-1 cells as described above.

**Day 1**. Development of 3D ‘mycobacteria-in-spheroid’ co-cultures

1. Prepare the THP-1 cell suspension as described in Workflow A above.

2. Prepare mycobacterial suspension as described in Workflow A.

Note: We used the *Mm* 1218 strain expressing GFP to infect freshly grown THP-1. This strain permitted 3D co-culture for a relatively longer duration when 50% of the culture medium in microwells was exchanged with fresh medium every seven days. We used a low MOI of 0.005 (optimal range: 0.005 to 0.008) to develop tuberculoma-imitative structures with cavity-like features. Optimizing MOI and extracellular matrix quantity is recommended when using different mycobacterial species or strains. We have yet to investigate the ability of additional *Mm* or *Mtb* strains in our collection to develop this feature in 3D cell culture.

3. Prepare 3D spheroid microplates for 3D co-cultures as described in Workflow A.

**Day 3**. Incorporation of the ECM solution in 3D ‘mycobacteria-in-spheroid’ co-cultures

1. Preparing the ECM solution

a. Add the required quantity of human collagen solution (3mg/ml) into a 15 ml sterile conical tube.

b. Prepare the ECM solution using human collagen and fibronectin solutions as described in Recipe 4.

Note: We have used a purified human collagen solution, VitroCol^®^. VitroCol collagen is naturally secreted from human neonatal fibroblast cells in the *in vitro* cell culture. The processed and pure form provided by the supplier was used. VitroCol is approximately 97% Type I human collagen, with the remainder comprising Type III collagen. Storage of VitroCol collagen at 2–8 °C is essential.

Critical: Do not freeze. While preparing the collagen mixture, gentle but thorough mixing and careful pH monitoring are crucial. Keep the mixture at 4 °C in the refrigerator or ice to prevent gelation. The mixture can be prepared a day in advance and stored at 2–8 °C overnight in the fridge to allow homogeneous pH adjustment of the solution. The fibronectin solution is added to the mixture at the end.

2. Adding the ECM solution

a. Add 30*−*50 µl of the ECM solution to each microwell containing the 3D co-culture on day 3.

Note Dispense the ECM solution slowly by slanting the micropipette tips against the side wall of the microwells. Control microwells without ECM addition can be kept, depending on the experiment’s objectives. We investigated the incorporation of different volumes of ECM solution, ranging from 5 to 50 µl, into 3D co-cultures. The workflow generates a tuberculoma-like structure with cavitary features in each microwell in 3D co-culture with 30−50 µl of ECM (See Results).

b. Place the microplates back into the CO_2_ incubator set at 37 °C with 5% CO_2_ and >95% humidity and continue the spheroid co-culture by incubating the microplates.

**Day 7 and day 14**. Addition of fresh medium

1. Add fresh 3D cell culture medium (perform as described above)

a. Carefully exchange 50% of the cell culture medium in the microwells with fresh, prewarmed (37 °C) medium.

Note: Exchange with a new medium is performed for co-cultures grown for more than 2 weeks.

b. Observe the growth of spheroid co-culture using a Cytation 5 imager or an inverted microscope (optional).

c. Continue the co-culture by incubating the microplates in the CO_2_ incubator.

Day 16 and day 21. Imaging and fluorescence intensity reading of microplates with 3D co-culture

1. Take fluorescence intensity reading and image the 3 spheroids

a. Place the microplate with lid into Cytation 5 set at 30 or 37 °C, with or without 5% CO_2._

b. Read the fluorescence intensity using optimized parameters as described above in **Table 1**, except that the excitation and emission wavelengths for GFP are 488 nm and 510 nm, respectively.

c. Capture images using the optimized protocol and parameters for brightfield and fluorescence imaging described above in **Table 2**, but use a GFP filter instead of a Texas Red filter.

d. Perform 3D image processing using the 3D montage imaging, stitching, and Z-stacking parameters described above (**Table 3**, **4**).

Note: After image capture, a Z-projection of the Z-stack images was generated using the focus-stacking algorithm described above in workflow A to create the final composite images.

e. Perform cellular analysis of 3D projected images using the parameters described above in **Table 5**, **6**.

#### 3.3.3 Workflow C for 3D bioplatform using purified human PBMC subsets

Note: This optimized workflow outlines the sequence of steps required to develop a bioplatform using co-cultures of purified human CD14–50%) of granuloma cells. However, the cellular composition can vary with early and late granulomas and granuloma forms (44). Primary monocytes that differentiate into macrophages constitute a small portion (approximately 10%) of the cells in human PBMC samples. Due to insufficient numbers of monocytes and macrophages in PBMC samples, which limit the continuous recruitment and maintenance of 3D *in vitro* granulomas, whole PBMCs generate small cellular aggregates rather than organized, dynamic granulomas. Therefore, purification and enrichment of CD14^+^ monocytes from PBMCs are critical steps in this workflow to develop the 3D tuberculoma model. In this workflow, 3D ‘mycobacteria-in-spheroid’ co-cultures are developed using MACS column-purified human primary CD14^+^ monocytes that differentiate into macrophages to simulate the formation of the core scaffold in human tuberculomas. The use of virulent *Mtb* strains is required. A flow-through unbound fraction generated during MACS column CD14^+^ monocyte purification contains CD14 microbead-unlabeled CD14^−^ cells (mainly lymphocyte subsets) from the PBMCs. This ‘lymphocyte-rich’ fraction is cultured separately for two days, washed, and then added to 3D spheroids generated from purified autologous CD14^+^ monocytes in 96-well microplates to simulate lymphocyte accumulation at the periphery of human tuberculomas with macrophage-rich centers. Granulomatous lesions (foci) develop in ‘mycobacteria-in-spheroid’ co-cultures even without adding this ‘lymphocyte-rich’ fraction. This bioplatform, employing innate and adaptive cell subsets purified from PBMCs, can be used to confirm’top hits’ identified in a primary screen in the THP-1 platform. The pre-experiment steps for this workflow include growing *Mtb* Erdman tdTomato as described above and identifying blood donors.

**Day 1**. Development of 3D ‘mycobacteria-in-spheroid’ co-culture

1. Separation of the PBMCs from whole human blood

Note: PBMCs can be isolated from anticoagulated human blood or “buffy coat” by density gradient centrifugation, for example, using Ficoll-Paque™. We used BD Vacutainer^®^ Cell Preparation Tubes (CPT™) for blood collection and PBMC separation. PBMCs were isolated following the manufacturer’s protocol (BD CPT Manual VDP40104–05 pg 1-2 (bdj.co.jp)). We used 200–250 ml of blood per human donor to isolate PBMCs and purify CD14^+^ monocytes. The isolated PBMCs and purified CD14^+^ monocytes were enough to develop five microplates.

Caution: Human whole blood may contain bloodborne pathogens, including HIV, HBV, and HCV. Blood from the prescreened donors was used, and universal precautions were taken to ensure safety.

a. Collect blood into BD Vacutainer^®^ tubes. Gently mix the blood by inverting the tubes 5 to 10 times before centrifugation.

Note: BD Vacutainer^®^ tubes should be stored at room temperature (18–25 °C) and labeled adequately for human donor identification.

b. Centrifuge tubes with a blood sample at room temperature in a horizontal rotor (swing-out head) for 30 min at 1500–1800× *g*.

c. Carefully pipette out the plasma without disturbing the white blood cell layer below immediately after centrifugation. Collect the cell layer containing mononuclear cells into a 50 ml conical tube. PBMCs from several CPT tubes can be pooled into one conical tube. Centrifuge the tubes at 1200× *g* for 15 min. Remove the supernatant without disturbing the cell pellet.

d. Lyse the RBCs by resuspending the pellet in 10 ml of 1× RBC lysis buffer for 5 min at room temperature.

e. Stop the lysis reaction by adding 25–30 ml of PBMC wash buffer (see Recipe 5), then gently mix the cell suspension with a pipette. Centrifuge the cells at room temperature at 275–300× *g* for 10 min. Carefully discard the supernatant.

f. Resuspend the cell pellet in the 20 ml PBMC wash buffer. Repeat the washing step thrice by centrifugation at 200× *g* for 10 min at 15–20 °C.

Note: These washing steps, performed at low-speed centrifugation, are required to remove platelets.

g. Resuspend the cell pellet in the PBMC wash buffer (20–25 ml buffer for PBMCs isolated from 200–250 ml of blood). Perform a cell count and assess cell viability using the trypan blue dye exclusion method.

Critical: Cell viability should be ≥ 95%. Dead cells may nonspecifically bind to MACS MicroBeads during purification of CD14^+^ monocytes.

h. Centrifuge cell suspension at 300× *g* for 10 min. Remove the supernatant. Adjust the final PBMC concentration to 1×10^7^ live cells in 80 µl of MACS buffer (see Recipe 6) to purify CD14^+^ monocytes.

Critical: A Buffer containing Ca^2+^ or Mg^2+^ is not recommended. Use a cold buffer (2–8 °C) to minimize nonspecific cell labeling.

2. Purification of CD14^+^ monocytes from PBMCs by positive selection.

Note: We purified CD14^+^ monocytes from the PBMCs by using the MACS technique and following the manufacturer’s protocol IM0001260.PDF (miltenyibiotec.com)

a. Add 20 µl of human CD14 MicroBeads to 10^7^ live PBMCs in 80 µl of MACS buffer and mix well by pipetting to magnetically label the CD14^+^ monocytes. Scale up all reagents and total volumes accordingly for higher cell numbers.

Note: MicroBeads are conjugated to monoclonal anti-human CD14 antibodies. Since CD14 lacks a cytoplasmic domain, it is considered that the antibody binding to CD14 does not trigger signal transduction in monocytes. Therefore, it does not affect the phagocytosis of *Mtb* bacilli.

b. Incubate for 15 min at 2–8 °C.

c. Wash cells by adding 1–2 ml of MACS buffer (Recipe 6) per 10^7^ cells and centrifuge at 300× *g* for 10 min. Remove the supernatant.

d. Resuspend up to 1×10^8^ cells in 500 µl of the MACS buffer.

e. Proceed to magnetic separation using LS columns.

Note: Choose an appropriate MACS Column and MACS Separator according to the number of total cells and CD14^+^ cells. For LS columns, the recommended sample size for leukocytes is 10^5^–10^8^ labeled cells in a total of 1×10^7^ to 2×10^9^ cells.

f. Place the LS column in the magnetic field of a MidiMACS separator attached to the MACS MultiStand.

g. Prepare the column by rinsing it with 3 ml of the MACS buffer (Recipe 6). Label collected effluent as ‘wash.’

h. Apply the cell suspension onto the column.

i. Collect the unlabeled cells that pass through, then wash the column with 3 ml of MACS buffer and collect the total effluent in a 50 ml conical tube. This unlabeled cell fraction contains lymphocytes and mononuclear cells, excluding CD14^+^ cells. Perform washing steps by adding 3 ml of MACS buffer three times. Label the tube as a ‘lymphocyte-rich’ fraction.

Note: Add a new buffer during washing steps only when the column reservoir is empty.

h. Remove the column from the separator and transfer it to a new 50 ml conical tube.

i. Pipette 5 ml of MACS buffer onto the column. Immediately flush out the magnetically labeled cells by firmly pushing the plunger into the column. Label the fraction as CD14^+^ monocytes.

j. Purify CD14^+^ monocytes from all isolated PBMCs from a donor. Centrifuge conical tubes containing purified CD14^+^ monocytes or the ‘lymphocyte-rich’ fraction at 300× *g* for 10 min.

k. Resuspend the ‘lymphocyte-rich’ fraction in 50 ml of PBMC growth medium (Recipe 2). Transfer cells to a 150 cm^2^ cell culture flask. Culture cells in a cell culture incubator set at 37 °C with 5% CO_2_ and >95% humidity for two days.

l. Resuspend the purified CD14^+^ monocyte pellet in 10 ml of PBMC growth medium (Recipe 2) and centrifuge at 300× *g* for 10 min. Remove the supernatant.

m. Wash the purified CD14^+^ monocytes three times by adding 10–15 ml of prewarmed (37 °C) 3D cell culture medium (Recipe 3) and centrifuging at 300× *g* for 10 min. Remove the supernatant. Resuspend purified monocytes in 10 ml of 3D cell culture medium.

n. Perform cell count and assess cell viability by the trypan blue dye exclusion method.

o. Adjust the cell concentration of CD14^+^ monocytes to 2×10^6^ live cells/ml using a 3D cell culture medium.

3. Development of 3D ‘mycobacteria-in-spheroid’ co-culture using CD14^+^ monocytes

a. As described above in Workflow A, develop ‘mycobacteria-in-spheroid’ co-cultures using CD14^+^ monocytes and *Mtb* Erdman (tdTomato) in the 96-well Corning 3D spheroid microplates.

Note:: Our experiments are routinely performed using 5,000 CFU of Mtb Erdman to infect 2×10^5^ live monocytes per microwell (MOI 0.025).

**Day 3**. Addition of lymphocytes and other CD14-negative mononuclear cells to the co-culture

1. Preparation and addition of ‘lymphocyte-rich’ cell subsets in the 3D microplate

a. Collect the ‘lymphocyte-rich’ cell fraction from the cell culture flask into a 50 ml conical tube.

Note: Use the cell scraper gently to release any adherent cells that have attached to the flask surface during the 2-day culture period.

b. Centrifuge the cell suspension at room temperature at 300× *g* for 10 min. Remove the supernatant. Resuspend the cell pellet in 15 ml of prewarmed (37 °C) 3D cell culture medium (Recipe 3).

c. Wash cells four times by adding 15 ml of prewarmed (37 °C) 3D cell culture medium. Centrifuge at 300× *g* for 10 min. Following the third wash, allow the cell suspension to rest at room temperature for 30 min before proceeding to the final centrifugation. After the last wash, resuspend cells in 10 ml of prewarmed (37 °C) 3D cell culture medium.

d. Perform a cell count and assess cell viability using the trypan blue dye exclusion method. Then adjust the cell concentration to 4×10^6^ live cells/ml using 3D cell culture medium.

e. Add 100 µl of cell suspension (4×10^5^ live cells) into each microwell without disturbing the spheroids formed by CD14^+^ monocytes. Periphery wells in the microplate are not used as described in the above section.

Note: Adding the ‘lymphocyte-rich’ cell fraction facilitates the recruitment of additional immune cell types, including those involved in adaptive immunity, in the monocyte- and macrophage-dominant spheroid core.

**Day 6**. Addition of test compounds or therapeutics

1. Carefully pipette 100 µl of the supernatant culture medium from the microwells, without disturbing the 3D spheroids. Next, as described above in Workflow A, add test compounds dissolved in the 50 µl solution.

**Day 12**. Fluorescence intensity reading and imaging to assess antitubercular drug efficacy

1. Perform fluorescence intensity reading and automated plate imaging as described in Workflow A for the THP-1 bioplatform.

Note: The workflow generates a complex, solid 3D tuberculoma-like structure that incorporates multiple adaptive and innate immune cell subsets within microwell co-cultures (See **Results**). The number of granulomatous lesions (foci) formed varies from donor to donor. Since primary human blood monocyte-derived macrophages lack self-renewal, granulomatous lesions may disintegrate relatively quickly after day 12 for some donors.

**Day 14**. Additional fluorescence intensity reading for microplates with *Mtb*-infected 3D culture

1. Perform additional fluorescence intensity reading on day 14 as described in Workflow A.

#### 3.3.4 Workflow D for the cryo-shelf-stable 3D bioplatform

Note: Cryopreservation is one of the most promising methods of long-term storage of cells and tissues. Therefore, we developed a cryostable bioplatform by freezing 3D co-cultures *in situ* in microplates at cryogenic temperatures. Three different time points, i.e., 30 min, 16 hr, and 72 hr post-*Mm* M (tdTomato) infection of THP-1 cells, were tested to determine the optimal time to freeze the microplates with 3D co-cultures. We also investigated three different freezing media (see Recipes 7a, b, and c). The optimized workflow and Results below describe the optimal freezing medium and time to freeze, detailing the steps required to develop a shelf-stable bioplatform in the 96-well 3D spheroid microplate. The pre-experiment steps for this workflow include growing *Mm* M tdTomato stocks and THP-1 cells as described above.

**Day 1**. Development and cryopreservation of 3D cell cultures

1. Preparing 3D spheroid microplates for co-culture and cryopreservation

a. Prepare THP-1 cell suspension and *Mm* suspension as described above in workflow A.

b. Resuspend the THP-1 cell pellet in the freezing medium (see Recipe 7a) to prepare a 2×10^6^ live cells/ml cell suspension.

Note: A greater number of THP-1 cells than workflow A is required to compensate for increased cell death during freezing and thawing.

c. Resuspend the *Mm* M (tdTomato) pellet in the freezing medium (see Recipe 7a) to prepare an 800−1000 CFU/ml suspension.

d. Dispense 100 µl of THP-1 cell suspension (1×10^5^ live cells) into each microwell except those on the periphery.

e. Add 100 µl of the mycobacterial suspension to each microwell except those on the periphery.

f. Mix thoroughly up and down three times without touching the microwell bottom to obtain a homogeneous suspension of THP-1 cells and mycobacteria.

g. Fill the periphery microwells with 250*−*300 µl of 3D cell culture media or sterile cell culture grade water using a multichannel pipette to avoid boundary effect.

h. Keep the microplates at room temperature for up to 30 min if freezing immediately.

Note: For ‘mycobacteria-in-spheroid’ co-cultures that were intended to be frozen after 16 or 72 hr, we incubated plates at 37 °C with 5% CO_2_ and >95% humidity for 16 or 72 hr (see below).

i. Place the microplates in cryo-boxes within 30 min post-infection or co-culture and transfer the 3D microplates into a -80 °C freezer.

Pause Point. Keep microplates in a -80 °C freezer for 12*−*72 hr.

j. Transfer the microplates to liquid nitrogen at -160 °C to -196 °C for long-term storage.

Pause Point. Transport the microplates to the liquid nitrogen storage facility on dry ice.

Note: For ‘mycobacteria-in-spheroid’ co-cultures intended to be frozen after 16 or 72 hr, we performed the co-culture in 3D cell culture medium in microplates as described in Workflow A. After 16 or 72 hr, the cell culture medium was carefully removed using a micropipette and replaced with a freezing medium. By 72 hr, the mycobacterial infection process in the spheroid is far advanced, with the formation of cell-to-cell junctions and adhesions. We avoided disturbing the early 3D spheroids when adding the freezing medium.

**Day of thawing**. Thawing of 3D co-culture and further culture on demand

1. Thawing of 3D co-culture and further culture on demand

a. Transport the microplates on dry ice from the liquid nitrogen storage facility to the cell culture laboratory.

b. Thaw frozen co-cultures in a CO_2_ incubator set at 37 °C with 5% CO_2_ and >95% humidity for 10 to 15 min.

Note: We removed microplate lids in a sterile incubator to facilitate quick thawing. Alternatively, microplates containing co-cultures can be carefully thawed on a platform in a water bath at 37 °C.

c. Add 50 µl of prewarmed (37 °C) 3D cell culture medium to each microwell once at least half of the freezing medium ice has visibly melted.

d. After complete thawing at 37 °C for 30 to 35 min, centrifuge the microplates at 430× *g* for 6 min.

e. Carefully pipette out the supernatant freezing medium, without disturbing the cell pellet.

Add fresh, prewarmed (37 °C) medium (200 µl per microwell) using a micropipette. Resuspend the cell pellet by pipetting up and down five times. Centrifuge the microplates at 430–450× *g* for 6 min. Perform this washing step three times to remove the freezing medium.

Critical: We did not centrifuge the microplates for ‘mycobacteria-in-spheroid’ co-cultures frozen 16 or 72 hr post-infection, when compact 3D structures (and cell-to-cell junctions) are already formed. Washing steps were performed by carefully adding pre-warmed medium (37 °C) without disturbing the 3D spheroid structures in microwells.

f. Resuspend the pellet in 200 µl of prewarmed (37 °C) 3D cell culture medium per microwell after the final wash.

g. Place the microplates in a CO_2_ incubator set at 37 °C with 5% CO_2_ and >95% humidity for further incubation, revival of the 3D co-culture, and formation of granuloma lesions (foci).

**Day 6 and day 12 post-thawing**. Addition of fresh 3D cell culture medium

1. Add prewarmed (37 °C) 3D cell culture medium (50 µl) without disturbing the 3D spheroids.

2. Return the microplates to the CO_2_ incubator to continue the growth of the 3D cell co-culture.

**Day 18 and day 21**. Fluorescence intensity reading and imaging

1. Perform the fluorescence intensity reading as described in Workflow A.

2. Perform imaging and lesion count as described in Workflow A.

Note: In a cryopreserved and revived ‘mycobacteria-in-spheroid’ co-culture, lesions (foci) develop relatively late compared to the freshly developed co-culture system. In this workflow, the optimal time to treat with investigational compounds is between days 14 and 18 post-revival for assessing drug efficacy.

## 4 Results

### 4.1 Optimization of infection dose in the 3D co-cultures

**Figure 3:**
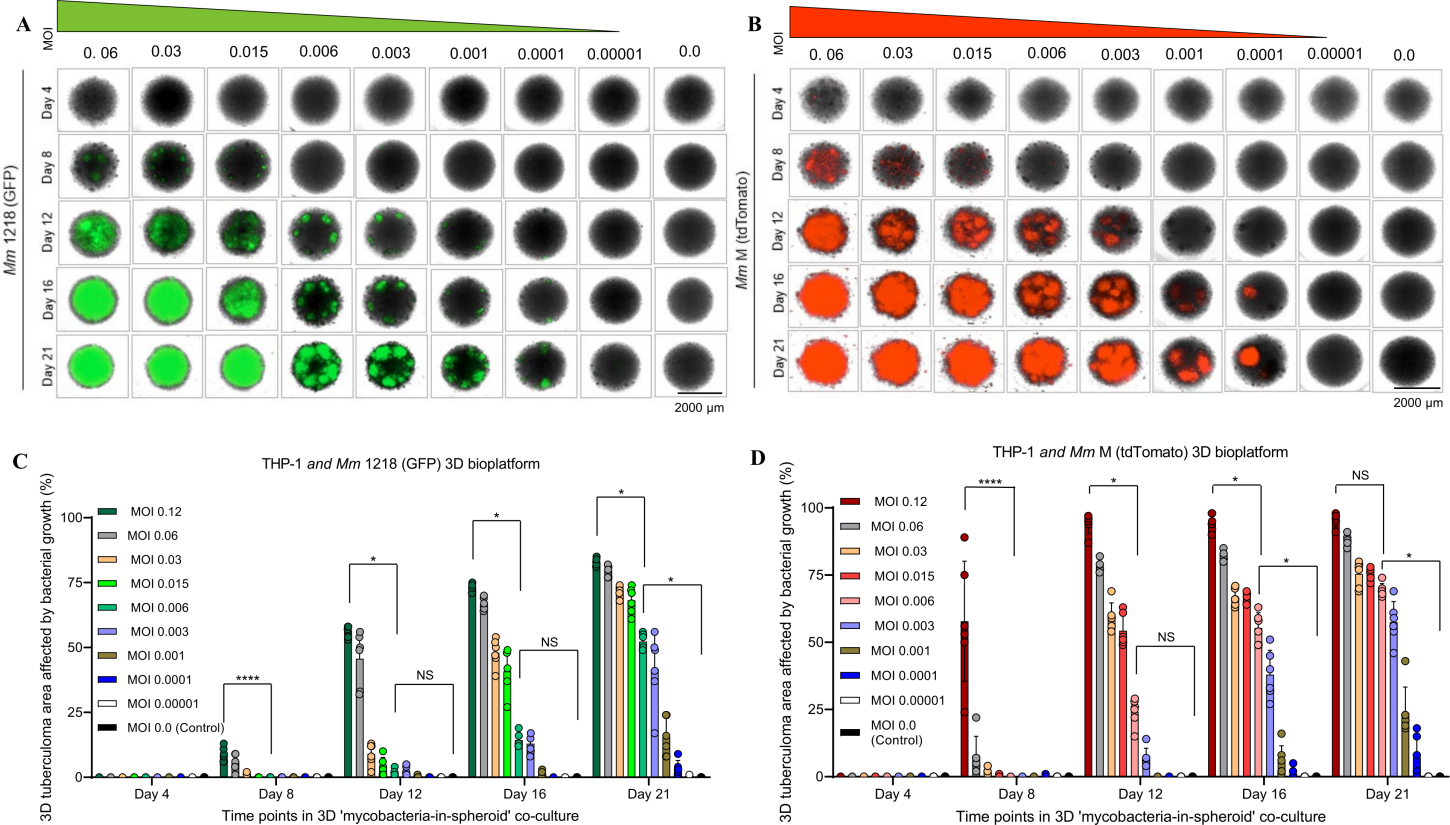
Investigating different multiplicity-of-infection doses of two fluorescent *M. marinum* strains in a ‘mycobacteria-in-spheroid’ co-culture workflow to optimize the 3D tuberculoma bioplatform. **(A, B)**. Kinetics of mycobacteria proliferation, granulomatous lesion (focus) formation, and the ensuing pathogenesis in ‘mycobacteria-in-spheroid’ co-cultures of THP-1 monocytes (1×10^5^) generated with eight different MOI doses (1 to 6000 CFU) of **(A)** *M. marinum* 1218 (GFP) and **(B)** *M. marinum* M (tdTomato) strain. Representative images are of one of the six 3D spheroids generated for each of the eight MOI doses or no-infection controls and were captured at five time points over three weeks of growth from one of the two experiments performed for each strain. Scale 2000 µm. **(C, D)**. Percent area affected by mycobacterial proliferation and granulomatous lesions (foci) in the 3D ‘mycobacteria-in-spheroid’ co-cultures generated using THP-1 monocytes (1×10^5^) and nine different increasing MOI doses (1 to 12,000 CFU) of **(C)** *M. marinum* 1218 (GFP) and **(D)** *M. marinum* M (tdTomato) strain. The circle represents the degree of pathogenesis within one 3D spheroid. The bar represents the average percentage of area affected by pathogenesis. The data are mean ± SD (n = 6 spheroids) from one of the two experiments. The co-culture using a low MOI (0.006; 600 CFU) of *M. marinum* strains results in an organized collection of granulomatous lesions (foci) with<50 and 75% of the 3D tuberculoma area affected by mycobacterial growth by day 12 and 21, respectively. The percentage of affected areas and the pathogenesis induced following a low MOI of 0.006 are compared with those caused by the standard MOI of 0.12 and with uninfected controls. * and **** indicate p< 0.05 and< 0.0001, respectively, using the Kruskal–Wallis test followed by Dunn’s *post-hoc* test. NS, nonsignificant.

We determined the optimal range of bacterial numbers required to infect monocytes in a 3D co-culture, without over-colonization over 2−3 weeks of incubation in 3D spheroids. The kinetics of mycobacterial proliferation, granulomatous lesion (focus) formation, and the resultant pathogenesis in the ‘mycobacteria-in-spheroid’ co-cultures of THP-1 monocytes (1×10^5^) using eight different MOI doses (1 to 6000 CFU) of *Mm* 1218 (GFP) and *Mm* M (tdTomato) were investigated over three weeks in early experiments (**Figures 3A–D**). Our findings revealed that a low MOI of 0.006 (600 CFU) resulted in organized granulomatous lesion formation with significantly less spheroid area affected by mycobacterial growth (*p* < 0.05) compared to the standard MOI of 0.12 by days 12 and 16. The optimal MOI for the *Mycobacterium* species and strains used in the 3D co-culture was determined by monitoring the dynamics of bacterial growth and granulomatous lesion formation within the 3D structures and by ascertaining the Z’-factor of the HTS assay performed in the resultant bioplatform (See **Data analysis and applications** below). The optimal MOI using *Mm* M (tdTomato) was determined to be 0.008 (i.e., 800 CFU per 10^5^ live THP-1 cells), within a range of 0.006–0.012. For the slow-growing *Mtb* strains H37Rv, Erdman, CDC 1551, and Beijing F2 expressing tdTomato, we found that a relatively higher MOI (0.012–0.05) was necessary for achieving an excellent (≥ 0.5) Z’-factor (See original study Sable et al., 2025) (45). Specifically, the optimal MOIs were 0.025 for *Mtb* H37Rv (tdTomato) and 0.05 for Erdman (tdTomato). Using a low physiological MOI ensures complete gathering of bacilli by aggregating THP-1 monocytes during 3D spheroid formation, preventing unwanted colonization outside the spheroid structures over the two weeks, without relying on aminoglycoside antibiotics, which are believed to kill only extracellular bacteria.

### 4.2 Fresh 3D co-cultures of human monocytes and pathogenic mycobacteria

**Figure 4:**
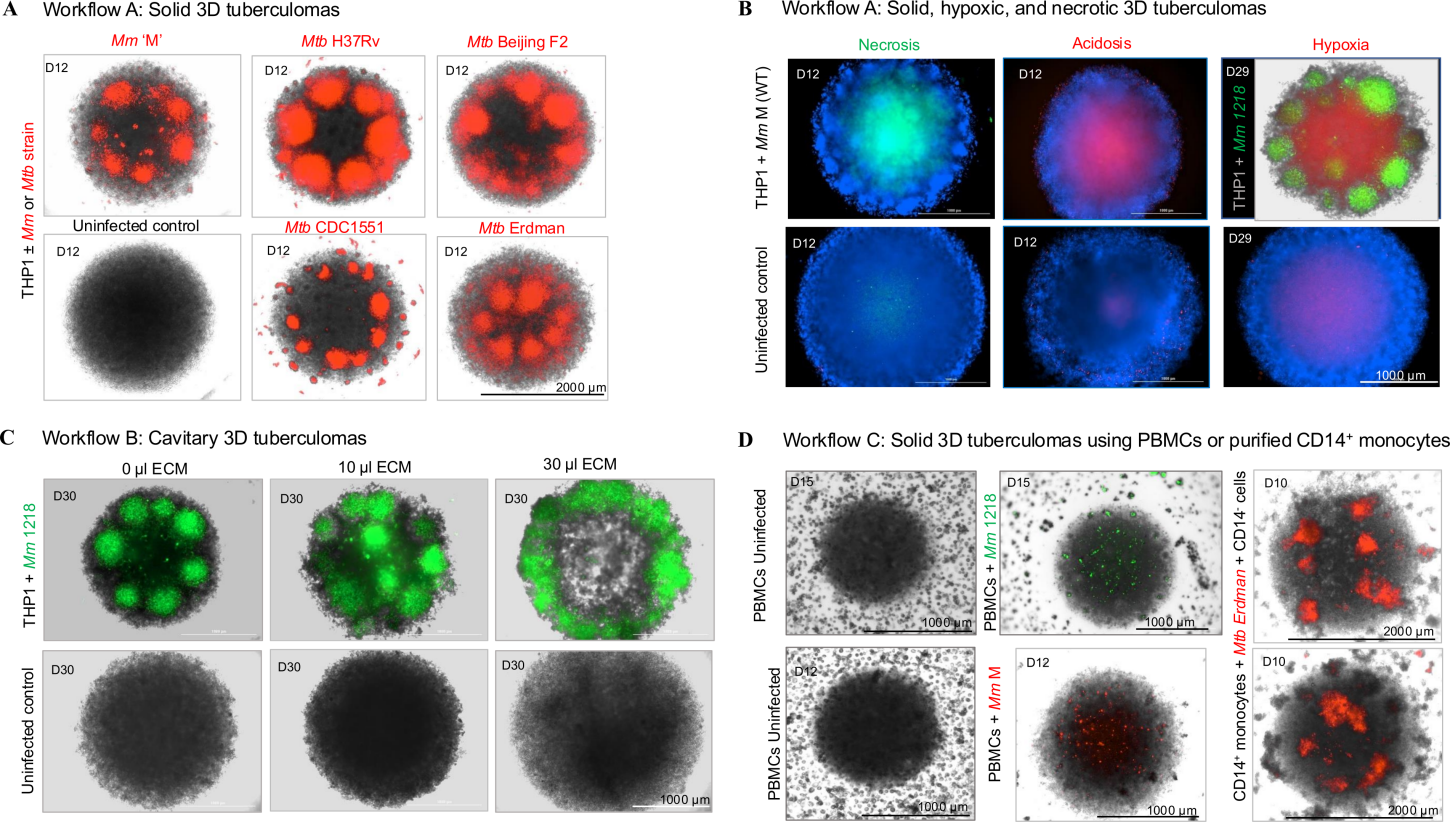
Tuberculoma forms developed using 3D ‘mycobacteria-in-spheroid’ co-culture workflows with freshly cultured human immortalized THP-1 cells or purified primary blood monocytes and pathogenic mycobacteria. **(A)** Solid tuberculoma-like structures generated following 3D co-cultures of freshly cultured THP-1 monocytes and pathogenic, *M. marinum* M, *M. tuberculosis* H37Rv, Beijing F2, CDC1551, or Erdman strain expressing tdTomato fluorescent protein and imaged on day 12. **(B)** Representative images of solid tuberculoma-like structures generated following 3D co-cultures of THP-1 monocytes and *M. marinum* wild-type or GFP stained for necrosis (green), acidosis (red), and hypoxia (red) using commercially available fluorogenic probes, as per the manufacturers’ instructions. Representative images of uninfected control spheroids are also shown. Blue counterstain indicates cell nuclei stained using Hoechst 33342 dye. **(C)** 3D tuberculoma-like structures generated following a modified 3D co-culture workflow of freshly cultured THP-1 monocytes and pathogenic *M. marinum* 1218 strain expressing GFP and incorporating human ECM comprised of type 1 human collagen and fibronectin (0 µl, 10 µl, or 30 µl). Cavitary features developed in the 3D tuberculomas but not in the control 3D spheroids with 30 µl of ECM. **(D)** Solid tuberculoma-like structures generated following 3D co-cultures of total human PBMCs and *M. marinum* 1218 (GFP) or *M. marinum* M (tdTomato) or following 3D co-cultures of purified CD14^+^ monocytes from human blood and *M. tuberculosis* Erdman supplemented with autologous lymphocyte-rich PBMC fraction. While 3D co-cultures of total PBMCs and *M. marinum* developed small granulomatous cellular aggregates, 3D co-cultures of purified CD14^+^ monocytes and *M. tuberculosis* Erdman developed larger, well-defined granulomatous lesions (foci). Representative images in **(A–D)** are from two or more independent experiments with at least three 3D spheroids.

The ‘mycobacteria-in-spheroid’ 3D co-culture workflow, involving freshly cultured human THP-1 monocytes and pathogenic mycobacterial strains *Mm* M, *Mtb* H37Rv, Beijing F2, CDC1551, or Erdman expressing tdTomato (Workflow A), successfully generated solid tuberculoma-like structures in microwells (**Figure 4A**). These 3D tuberculoma-imitative structures encompassed an organized conglomeration of granulomatous lesions (foci) and exhibited critical attributes, including hypoxia, necrosis, and acidosis, in their cores (**Figure 4B**). In a modified 3D co-culture of THP-1 monocytes and *Mm* 1218 strain expressing GFP and supplemented with human ECM solution (Workflow B), 3D tuberculoma-like structures with cavity-like features were produced (**Figure 4C**). The volume of the ECM solution and the MOI of *Mm* 1218 used influenced the formation of cavitary features. Specifically, 30 µl of ECM solution induced the development of cavity-like features in the 3D co-cultures, but not in the control 3D spheroids. Whereas lower volumes of ECM (<25 µl) or very low MOI (50 CFU) resulted in no cavity-like feature formation. In experiments with fluorescent *Mm* strains and human whole PBMCs, which contain fewer than 10% monocytes, small granulomatous aggregates were formed in 3D spheroids (**Figure 4D**) due to the insufficient number of monocyte-derived macrophages to sustain granuloma organization. However, 3D co-cultures of MACS-purified primary CD14^+^ monocytes (2×10^6^/ml) with *Mtb* Erdman (tdTomato), and supplemented with CD14^−^ PBMC subsets (Workflow C), developed larger, organized granuloma lesions, comparable to those formed using immortalized THP-1 monocytes and pathogenic *Mm* or *Mtb* strains. The solid 3D tuberculoma-like structure generated using this workflow thus contains both innate and adaptive immune cell types present in human PBMCs.

### 4.3 Cryopreserved 3D co-cultures

**Figure 5:**
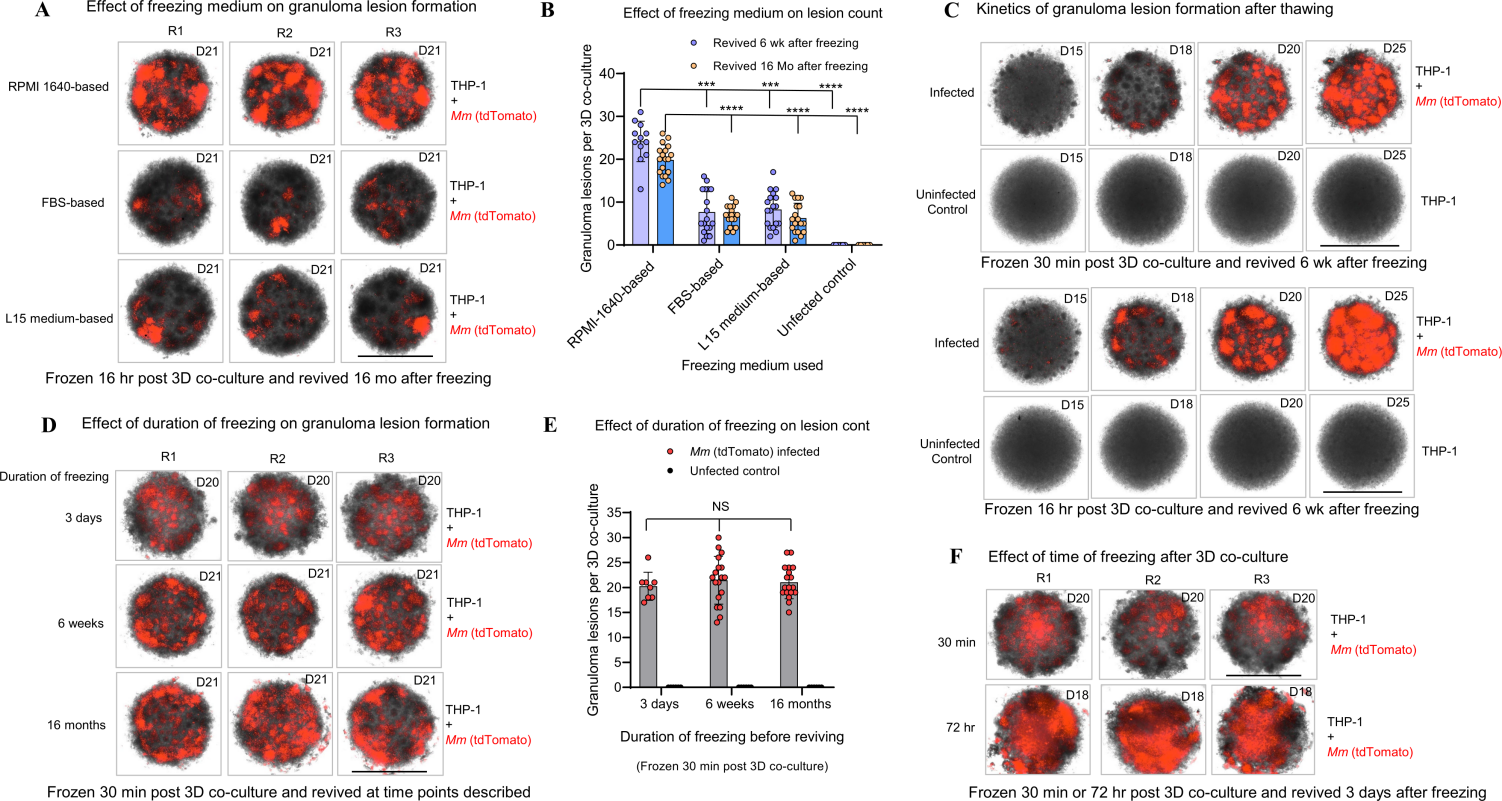
Solid tuberculoma-like structure formation following cryopreservation and revival of 3D co-cultures using THP-1 monocytes and *M. marinum* (M) **(A, B)** Effect of three different freezing media on the granuloma formation in the 3D co-cultures. Significantly more granulomatous lesions (foci) developed in 3D co-cultures that were frozen 16 hours post-co-culture in the RPMI 1640-based freezing medium compared to the FBS-based or L15 medium-based freezing medium and then revived 6 weeks or 16 months after freezing. **(C)** Kinetics of granulomatous lesion formation after thawing frozen 3D co-cultures. A comparable trend in granulomatous lesion formation was observed in 3D co-cultures that were frozen 30 minutes or 16 hours post-co-culture in the RPMI 1640-based freezing medium and then revived 6 weeks after freezing. **(D)** and **(E)** Effect of duration of freezing on the granulomatous lesion formation. No significant difference in granuloma count was found in the 3D co-culture frozen in RPMI 1640-based freezing medium 30 minutes post-co-culture and revived after 3 days, 6 weeks, or 16 months of freezing. **(F)** Effect of time of freezing after 3D co-culture on organized granuloma formation. Numerous well-defined granulomatous lesions developed in the 3D co-cultures that were frozen 30 minutes or 16 hours post-co-culture (in **(A, C)**), compared to 72 hours post-co-culture, in the RPMI 1640-based freezing medium and then revived after 3 days of freezing. Scale 2000 µm. Representative images and data (mean ± SD) are from one to three independent experiments (n = 6–16 spheroids/group). Circles in histograms represent the number of granulomatous lesions (foci) per 3D co-culture. ***p< 0.001 and ****p< 0.0001 using the Kruskal–Wallis test with Dunn’s *post-hoc* test. NS, nonsignificant.

We evaluated three freezing media—3D cell-culture medium with 5% DMSO, heat-inactivated FBS with 5% DMSO, and Lebovitz’s L-15 medium with cryoprotective agents—to identify the most effective option for cryopreserving human THP-1 monocyte and *Mm* M (tdTomato) 3D co-cultures (Workflow D). Additionally, we tested three time points (30 min, 16 hr, and 72 hr post-infection) to determine the optimal timing for freezing the microplates with 3D co-cultures. The best results, characterized by organized granulomatous lesion (foci) formation after thawing and revival, were achieved when 3D co-cultures were frozen between 30 min and 16 hr post-infection using the 3D cell culture medium with 5% DMSO for cryopreservation (**Figures 5A−F**). In contrast, more extended co-culture periods, exceeding 72 hr post-infection before cryopreservation, resulted in suboptimal outcomes after revival, likely due to the formation of solid 3D structures, cell-to-cell junctions, and nascent lesions.

### 4.4 Data processing and analysis

The 3D cell culture workflows using human THP-1 cells or primary CD14^+^ blood monocytes with pathogenic mycobacteria, as described here, were employed in our original study (45). This approach developed 3D structures analogous to solid tuberculomas without the need for ECM embedding, artificial scaffolds, or magnetic levitation. In proof-of-concept experiments, the resulting solid tuberculomas in 96-well format were used to screen a custom library of known potential HDT chemical compounds. The efficacy of the compounds was evaluated by their ability to inhibit bacterial burden and granulomatous lesions. Mycobacterial load was determined by measuring the fluorescence intensity of red-fluorescent *Mm* or *Mtb* strains expressing tdTomato or by counting CFU on Middlebrook 7H10 agar. This was done by disrupting spheroids and lysing the cells with Triton X-100 (0.1%) for 10 minutes, then plating the resulting lysate. Granulomatous lesion counts in the 3D structures were obtained through automated imaging and cellular analysis of stitched and Z-stacked images, with manual counts performed by three readers to ensure quality control against Gen5 calculations.

#### 4.4.1 Application in drug screening assay

**Figure 6:**
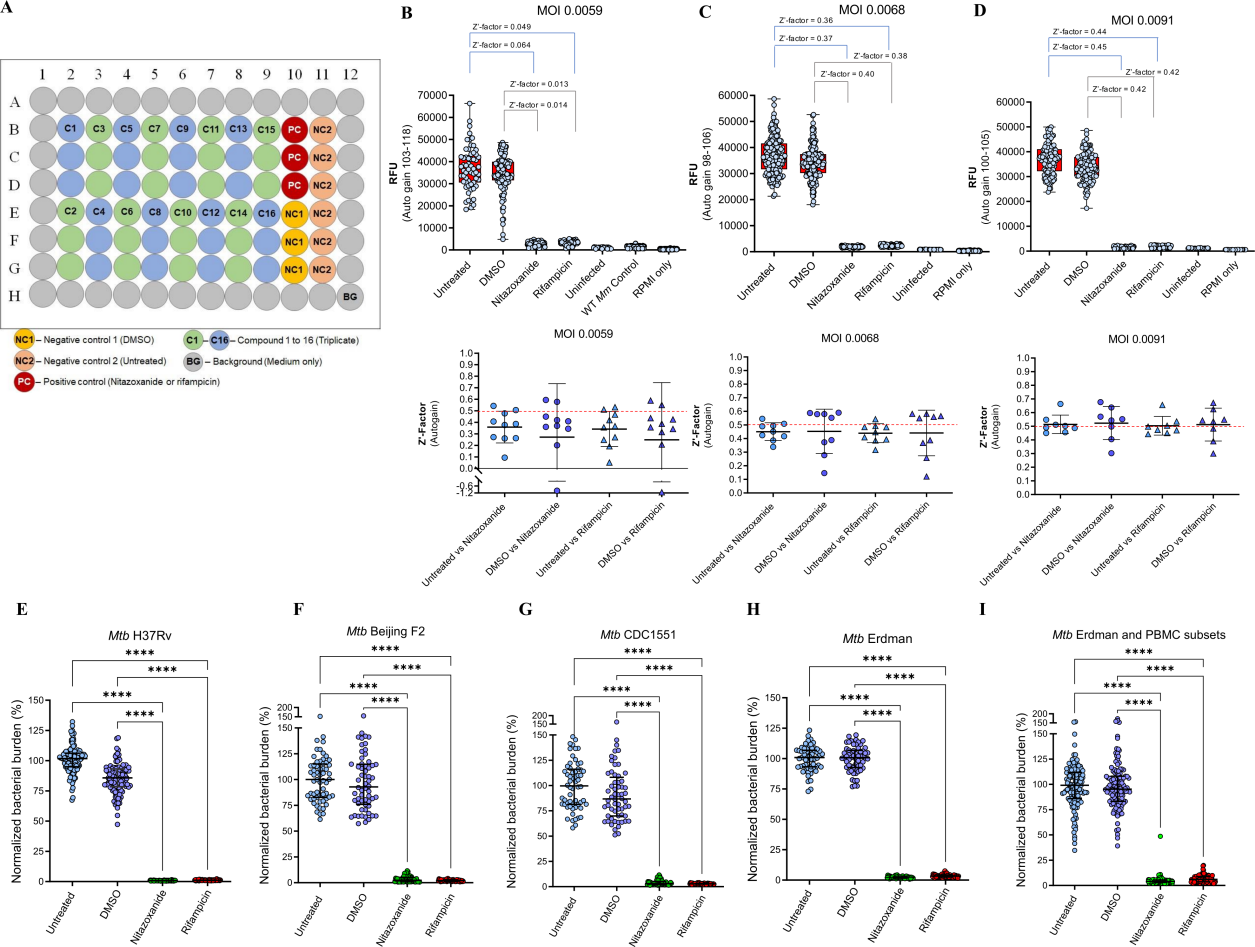
High-throughput screening compatibility and assay quality in 3D tuberculoma bioplatform. **(A)** Plate map showing the typical locations of control and test wells on a 96-well plate. In a primary screen, 16 compounds can be tested in triplicate per plate. **(B–D)** The effect of infection dose (MOI) on the Z’-factor of the drug screening assay in the 3D tuberculoma bioplatform developed using THP-1 monocytes with varying infection doses of *M. marinum* M (tdTomato). Three different MOI doses, 0.0059 **(B)**, 0.0068 **(C)**, and 0.0091 **(D)**, were tested. The upper panels show the Z’ factor of the screening assay across eight to ten 96-well plates per MOI experiment, including both negative and positive controls in each plate. Relative fluorescence units (RFU), which reflect bacterial burdens, are shown as box plots with whiskers (minimum to maximum) capturing all data points, with circle symbols indicating RFU levels in individual wells. Fluorescence intensity was measured using auto-gain, and the range of auto-gain values for the plates used in the experiment is shown. Untreated, DMSO, Nitazoxanide, and Rifampicin (in **B–D**) refer to 3D *in vitro* tuberculomas of THP-1 monocytes infected with *Mm* M tdTomato, either untreated (no drug, medium only) or treated with DMSO, nitazoxanide (20 µM), or rifampicin (1 µg/ml). WT *Mm* Control refers to 3D co-cultures of THP-1 monocytes infected with the wild-type *Mm* M strain (nonfluorescent). Uninfected (3D cell culture of THP-1 cells alone) and RPMI only (wells containing only RPMI-1640 medium without cells or bacteria) serve as additional controls. The lower panels show Z’ factors in individual plates of the bioplatform assay, where each symbol represents the Z’ factor in one microplate. The assay using MOI 0.0091 demonstrated excellent to good assay quality (average Z’ factor ≥ 0.5, indicated by the dotted red line). **(E–H)** Show the reduction in bacterial burden following treatment with nitazoxanide (20 µM) and rifampicin (1 µg/ml) in 3D *in vitro* tuberculomas of human THP-1 monocytes infected with *Mtb* H37Rv (MOI 0.025), Beijing F2 (MOI 0.035), CDC1551 (MOI 0.05), or Erdman (MOI 0.05), expressing tdTomato, or **(I)** in 3D *in vitro* tuberculomas of human CD14^+^ monocytes infected with *Mtb* Erdman (MOI 0.025), expressing tdTomato and supplemented with CD14^−^ subsets (lymphocyte-rich PBMC fraction). Filled circles represent bacterial burden in individual 3D *in vitro* tuberculomas; data are from 2–4 experiments per *Mtb* strain **(E–H)** or from 4 experiments using PBMC subsets from 4 healthy donors **(I)**. n = 61–123 *in vitro* tuberculomas per untreated or DMSO control, and 49–98 tuberculomas per nitazoxanide and rifampicin. A horizontal line with error bars indicates the median with the interquartile range. ****p< 0.0001 using the Kruskal–Wallis test with Dunn’s *post-hoc* test.


The bioplatform enables serial quantification of drug efficacy *in situ*, as measured by bacterial burden reduction, using a fluorescence plate reader. Additionally, it facilitates the imaging of granulomatous lesion resolution using an automated cell imaging system, as described in the workflows above. The efficacy of test compounds, in terms of reduction or increase in bacterial burden, was determined by treating 3D co-cultures with individual test compounds in at least triplicate wells (termed test wells). Wells treated with drug diluent or carrier (i.e., DMSO) and cell culture medium alone without the drug (negative controls) and the antibiotic rifampicin or the experimental HDT drug nitazoxanide (positive controls) were kept in each microplate (see plate map, **Figure 6A**). A pre-defined plate map was used to identify drugs added to microwells. Each microplate contained 3–6 DMSO or carrier-treated wells, 3–6 untreated (no-drug) wells, 3 rifampicin or nitazoxanide-treated wells, and 36 peripheral wells with medium without cells (background) as controls. The normalized reduction of bacterial burden (%) in test compound-treated wells was calculated using the formula below, where Ft is the fluorescence intensity of test compound-treated wells, and Mean Fu is the average fluorescence intensity of untreated (no-drug) or DMSO-treated control wells (n = 3–6 tuberculomas).

$$\text{Percent bacterial burden reduction}=\;\frac{\left({\text{F}}_{\text{t}}-{\text{Mean F}}_{\text{u}}\right)}{{\text{Mean F}}_{\text{u}}}$$

#### 4.4.2 Quality and suitability of assay for use in HTS

The bioplatform assay reliably and consistently detected changes in mycobacterial burden, granuloma counts, and lesion size across microwells on multiple plates per experiment after treatment with test compounds. With rifampicin and nitazoxanide, we observed a consistent reduction in bacterial burden measurements (**Figures 6B–D**), achieving a Z’-factor of >0.4 across microwells and an average Z’-factor of ≥0.5 among several plates (n = 8) when an MOI of 0.009 of *Mm* M (tdTomato) was used in the 3D cell culture microplates (**Figure 6D**). This indicates that the assay is of acceptable to excellent quality and robustness for HTS applications at this MOI (refer to the statistical analysis section for Z’-statistics). An MOI below 0.006 resulted in a Z’-factor<0 in one out of ten plates, suggesting that this MOI is suboptimal for screening assays. Please also refer to our original study (45) for additional information on assay quality (Z’-factor >0.5) for the *Mtb* H37Rv assay, the optimal MOI for *Mtb* strains, and other aspects of data processing and analysis during the screening of potential therapeutics. Rifampicin and nitazoxanide also effectively reduced the bacterial load in 3D tuberculomas of *Mtb* H37Rv, Beijing F2, CDC1551, or Erdman expressing tdTomato compared with untreated (no-drug) and DMSO controls (**Figures 6E–I**).

#### 4.4.3 Other applications

Beyond drug screening applications, in the original study (45), we demonstrated the utility of the tuberculoma bioplatform to assess the effects of test compounds simultaneously on host-cell viability, the type of cell death induced, and the potential innate immune mechanisms of action in 3D microenvironments *in situ*. We also demonstrated the utility of this 3D model system for investigating the effects of biologics and biosimilars on granuloma formation and maintenance of granuloma architecture, as well as for deciphering early *Mtb*−host interactions in solid or cavitary tuberculoma milieus.

### 4.5 Validation of protocol

#### 4.5.1 Uniformity and reproducibility of bioplatform

**Figure 7:**
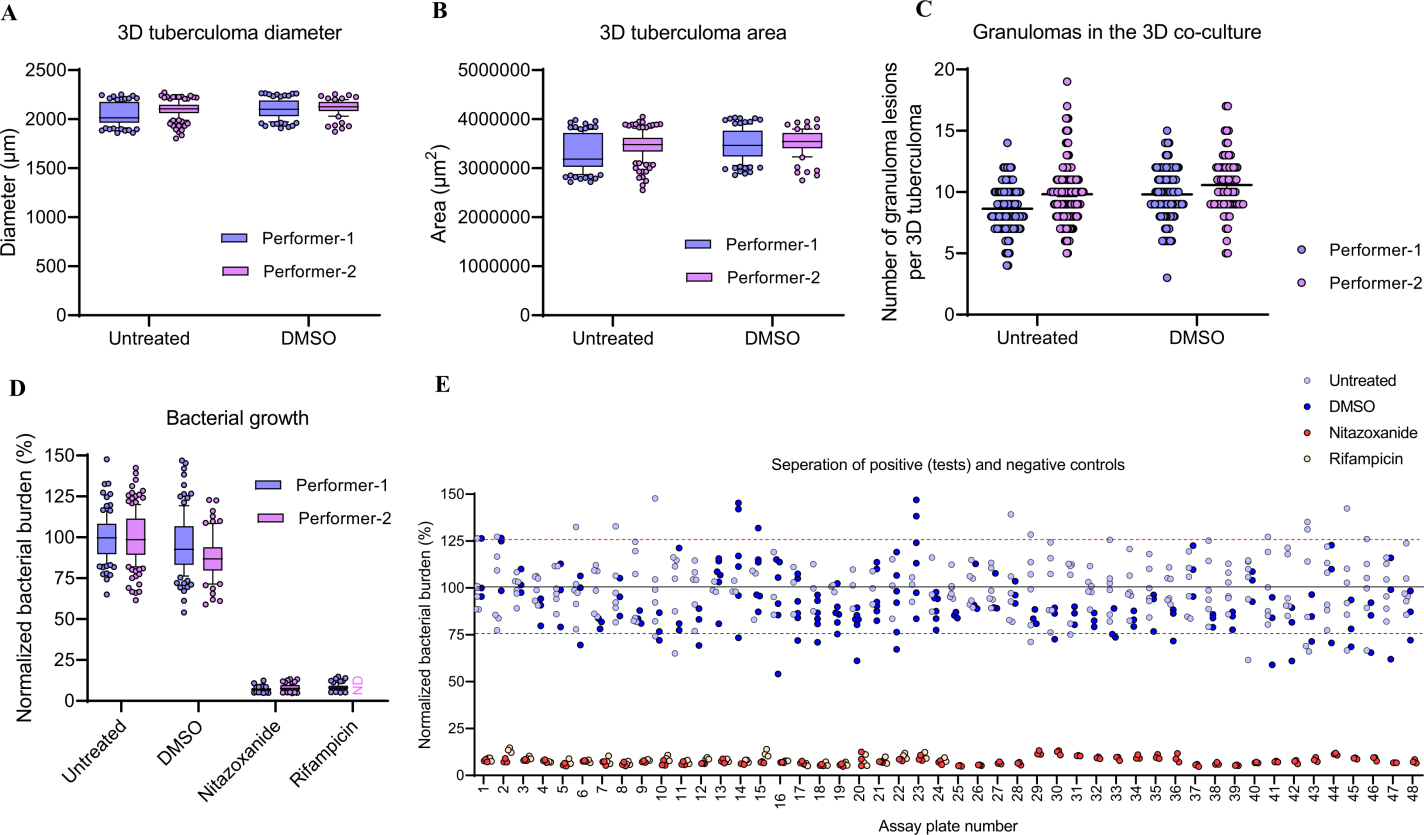
Uniformity and reproducibility of 3D *in vitro* tuberculomas and bioplatform. **(A-D)** Consistency in the diameter **(A)**, area **(B)**, granulomatous lesion count **(C)**, and bacterial burden **(D)** in the solid 3D tuberculoma-like structures developed in 96-well microplates by two separate assay performers using freshly cultured THP-1 monocytes and *M. marinum* M tdTomato. **(E)** Separation of bacterial burden in nitazoxanide (20 µM) or rifampicin (1 µg/ml)-treated (positive controls) and untreated or DMSO-treated (negative controls) 3D tuberculomas. Data in box plots (whiskers: 10–90 percentile) in **(A–D)** are from a total of 648 *in vitro* tuberculomas (72–144 tuberculomas per treatment) from 48 different assay plates included in 12 independent experiments (6 experiments per performer) of chemical compound library screenings using the bioplatform assay. Circles represent a response per 3D tuberculoma. The parameters in the 3D tuberculomas developed by the two performers were not significantly different, as determined by the Mann-Whitney test. ND, not done. Bacterial burden data in **(E)** are from 3–6 *in vitro* tuberculomas per treatment across 48 cell culture plates (24 plates per performer), developed for screening potential pathogen-targeted and host-directed compounds using the 3D tuberculoma bioplatform. Rifampicin was not tested in plates 25–48 developed by performer 2.

We assessed the efficiency, homogeneity, and reproducibility of 3D tuberculoma-like structures formed in the 96-well platform across multiple microplates and independent experiments. The diameters and areas of 3D tuberculoma-like structures generated across different microplates and batches developed by two performers were measured to determine efficiency, uniformity, and reproducibility. We also analyzed the bacterial burden, the number of granulomatous lesions (foci) produced in the 3D spheroids, and the percentage area of the tuberculoma-like structures affected by mycobacterial growth and lesions. The bioplatform reliably generated 3D tuberculoma-like structures each time. Co-culturing human THP-1 monocytes with fluorescent *Mm* or *Mtb* strains at optimal MOI in Corning^®^ ULA 3D spheroid plates consistently produced 3D tuberculoma-like structures with an organized collection of granulomatous foci across all microwells and experiments. Similar tuberculoma-like structures were observed when human CD14^+^ monocytes from healthy donors were co-cultured with *Mtb* Erdman tdTomato at the optimal MOI. The resulting 3D structures of THP-1 monocytes exhibited minimal variability (both within and between batches) in size distribution, mycobacterial growth, and granulomas encompassed by individual tuberculoma structures (**Figure 7A–E**). Their uniformity, reproducibility, and ease of development render them ideal for HTS applications.

#### 4.5.2 Other validations

In the previous sections, we have provided evidence that the developed protocol is highly robust and reproducible. The number of replicates, controls, and the statistical tests used for validation are described above and in the individual figure legends. For additional details about the characterization of the 3D tuberculoma model developed using primary or immortalized human monocytes, including attributes such as autophagy, inflammasome activation, hypoxia, necrosis, and lysosomal acidification in 3D structures, please refer to the Main and Supplementary Figures in the original study (45). For information about how cell culture plate type and culture conditions were selected for 3D *in-vitro* tuberculoma model generation and for more details about the workflow incorporating human ECM to generate 3D tuberculoma-like structures with cavity-like features, please refer to the original study (45). Using whole-mount immunostaining *in situ* in the original study, we identified key tuberculous granuloma and epithelioid macrophage markers, including E-cadherins, ICAM-1, and γ-catenin, in early granulomatous aggregates of infected spheroids, indicating that these cellular aggregates (foci) are genuine nascent granulomas. Additional information about the validation of the utility and the HTS-compatibility of the 3D tuberculoma bioplatform used for screening antimicrobials and HDT compounds within a custom chemical compound library during the proof-of-concept experiments is provided in the original study (45).

## 6 Troubleshooting

Troubleshooting information is provided as **Supplementary Table 10**.

## 7 Discussion

The advanced cell culture systems that co-culture pathogenic mycobacteria with human immune cells, yielding 3D *in vitro* granuloma models and bioplatforms with HCS capabilities (25–27, 29–31, 33), represent highly innovative approaches and have fueled hopes of discovering clinically relevant pathogen-targeting and host-directed therapeutics. However, existing 3D human *in vitro* granuloma models face challenges regarding ease of generation, throughput, scalability, reproducibility, shelf stability, and screening efficiency. Additional impediments to their utility include technical complexities, difficulties in cell sourcing, limited ability to study granuloma dynamics over time, challenges in retrieving cells for analysis, and limited representation of the microenvironments. A simplistic but high-fidelity bioplatform is highly desirable (33). Yet, the minimum composition and optimal culture conditions for such a facile system for HTS applications, without compromising TB granuloma formation and tuberculoma attributes, remain unresolved. The observation that facile 3D co-culture of human primary CD14^+^ monocytes or immortalized THP-1 cells with pathogenic mycobacteria, as minimum essential components, generates crucial features and structures analogous to tuberculomas in the workflows described here is striking. This simplistic ‘mycobacteria-in-spheroid’ 3D co-culture model, in a 96-well format, transforms monocytes into epithelioid macrophages and forms tuberculoma-like structures with a collection of granuloma lesions (foci). This confirms findings in animal models that tuberculous granuloma formation occurs in the absence of adaptive immunity, in the sole context of innate immunity (2, 41, 46), and provides an HTS-compatible platform with a range of tuberculoma microenvironments for studying host-pathogen interactions and therapeutic screening.

Despite using spheroid co-cultures to model TB granulomas and generating 3D cellular structures with hypoxia and necrosis, tuberculoma-like features, including an organized collection of granulomas or cavitation, have not been reported in available models (29, 47, 48). The formation of tuberculoma-like structures and traits in our model required unique spatial features in a culture plate, defined culture conditions, and a specific timeframe. 3D spheroid models are ECM-anchorage- or scaffold-independent systems. Macrophages and ancillary cells can contribute to ECM production in 3D cultures. Adding exogenous ECM into the 3D granuloma model prolongs macrophage survival compared to monolayer cultures (26). However, exogenous ECM can reduce granuloma size and limit *Mtb* proliferation (37) and may potentially introduce variability due to differences in ECM composition and properties. The facile 3D model developed here forms solid tuberculoma-like structures with hypoxic and necrotic cores, without requiring exogenous ECM, collagen embedding, or polymer-encapsulation of macrophages. This approach reduces the system’s complexity while preserving the pliability, scalability, and reproducibility of the bioplatform, ultimately improving throughput.

As described above, the 3D tuberculoma bioplatform has several advantages over existing systems. These include ease of development and use, robustness, self-assembly, cryo-stability, lower costs, increased throughput, and efficiency. The bioplatform also allows real-time monitoring of mycobacterial burdens, granulomatous lesions (foci), host cytotoxicity, cavitary transformation, and multiparameter tracking of additional bacterial and host-cell physiological attributes *in situ*. The cryopreserved version of the tuberculoma bioplatform can be frozen for future use and revived as needed. The human-relevant model could offer anti-vivisection humane benefits through minimizing animal use in TB research. The estimated cost of performing one experiment is $65 using a 96-well 3D tuberculoma bioplatform (produced in a Corning ULA Spheroid microplate) to test 16 compounds in triplicate. This expense is comparable to that of a 2D cell culture experiment with a 96-well Corning ULA 2D flat-bottom microplate. In contrast, screening a single test compound in a mouse model of TB (6 mice per group) in an ABSL-3 laboratory over a month costs approximately $1000. Therefore, employing the 3D tuberculoma bioplatform for screening large compound and genetic libraries is very economical, ethical, and effective, while reducing animal use.

One limitation of the 3D tuberculoma model and workflows described here is the absence of relevant ancillary cells present in human tuberculous granulomas, such as granulocyte subsets and non-hematopoietic cells, including fibroblasts, epithelial cells, and endothelial cells. Consequently, our model system was unable to investigate the contributions of neutrophils and stromal cells in the formation of necrotizing and caseous tuberculomas and cavitary transformation. To further improve our model, we have successfully incorporated these cell types into the THP-1 bioplatform as human cell lines, demonstrating the system’s flexibility while investigating the effects of their addition on granuloma organization in preliminary experiments (data not shown). Since the tissue-like 3D structures are relatively large (>2100 µm in diameter), we used 2.5× magnification on the Cytation 5 to visualize the entire structure during automated plate imaging. However, at this magnification or when viewing individual cross-sectional slices, subcellular details and the precise locations (intracellular or extracellular) of mycobacteria remained unclear. Our ongoing imaging studies, including tissue clearing to visualize 3D tuberculoma-like structures and higher-magnification imaging, along with flow cytometry of isolated cells, will help contextualize the fluorescence results within granulomatous foci and individual cells.

Compared with other available 3D human *in vitro* granuloma models, such as those using polymer encapsulation or matrix embedment, bio-electro-spraying, and magnetic levitation, the methodology described here is straightforward and requires no specialized instruments or complex materials for development. It provides the tool for investigating heterogeneous granuloma responses to TB vaccines and immunotherapies. It has advantages over the newest 3D systems that facilitate investigations of host-pathogen interactions but lack granuloma formation or 3D tuberculoma features (49, 50). It can be used in siRNA and CRISPRi library screens to identify druggable host targets in granulomas. Beyond HTS application for identifying potential HDT compounds and antimicrobials, the bioplatform might allow the development of personalized medicine and therapies using engineered primary human cells, for example, the chimeric antigen receptor (CAR)-macrophages, NK cells, and T cells of patients with difficult-to-treat mycobacterial infections or granulomatous diseases other than TB. The bioplatform can be modified to investigate foreign-body granulomas, non-infectious granulomatous disorders (for example, sarcoidosis, Crohn’s disease, granulomatosis with polyangiitis), and infectious granulomatous diseases, such as those caused by other mycobacteria (e.g., leprosy), bacteria (e.g., brucellosis, cat scratch disease, Whipple’s disease, and *Chromobacterium violaceum* granulomas), fungi (e.g., coccidioidomycosis, cryptococcosis, and histoplasmosis), parasites (e.g., schistosomiasis, leishmaniasis, and toxoplasmosis), and viruses (e.g., rubella-induced granulomas).

## Data Availability

The original contributions presented in the study are included in the article/**Supplementary Material**. Further inquiries can be directed to the corresponding author.

## Author disclaimer

The findings and conclusions in this publication are those of the authors and do not necessarily represent the official position of the CDC, its affiliated organizations, or those of the publisher, editors, and reviewers. In addition, the trade names used in the study are for identification only and do not constitute an endorsement by the U.S. Department of Health and Human Services, the Public Health Services, or the CDC. Any product that may be evaluated in this article, or claim made by its manufacturer, is not guaranteed or endorsed by the publisher.
